# Cytochrome *b*_*5*_ reductase and the control of lipid metabolism and healthspan

**DOI:** 10.1038/npjamd.2016.6

**Published:** 2016-05-12

**Authors:** Alejandro Martin-Montalvo, Yaning Sun, Alberto Diaz-Ruiz, Ahmed Ali, Vincent Gutierrez, Hector H Palacios, Jessica Curtis, Emilio Siendones, Julia Ariza, Gelareh A Abulwerdi, Xiaoping Sun, Annie X Wang, Kevin J Pearson, Kenneth W Fishbein, Richard G Spencer, Miao Wang, Xianlin Han, Morten Scheibye-Knudsen, Joe A Baur, Howard G Shertzer, Placido Navas, Jose Manuel Villalba, Sige Zou, Michel Bernier, Rafael de Cabo

**Affiliations:** 1 Translational Gerontology Branch, National Institute on Aging, National Institutes of Health, Baltimore, MD, USA; 2 Centro Andaluz de Biología del Desarrollo, and CIBERER, Instituto de Salud Carlos III, Universidad Pablo de Olavide-CSIC, Sevilla, Spain; 3 Departamento de Biología Celular, Fisiología e Inmunología, Facultad de Ciencias, Universidad de Córdoba, Campus de Excelencia Internacional Agroalimentario, Córdoba, Spain; 4 Graduate Center for Nutritional Sciences, University of Kentucky, Lexington, KY, USA; 5 Magnetic Resonance Imaging and Spectroscopy Section, National Institute on Aging, National Institutes of Health, Baltimore, MD, USA; 6 Diabetes and Obesity Research Center, Sanford-Burnham Medical Research Institute, Orlando, FL, USA; 7 Laboratory of Molecular Gerontology, National Institute on Aging, National Institutes of Health, Baltimore, MD, USA; 8 Department of Physiology, Institute for Diabetes, Obesity, and Metabolism, University of Pennsylvania, Philadelphia, PA, USA; 9 Department of Environmental Health, University of Cincinnati College of Medicine, Cincinnati, OH, USA

## Abstract

Cytochrome *b_5_* reductases (CYB5R) are required for the elongation and desaturation of fatty acids, cholesterol synthesis and mono-oxygenation of cytochrome P450 enzymes, all of which are associated with protection against metabolic disorders. However, the physiological role of CYB5R in the context of metabolism, healthspan and aging remains ill-defined. We generated CYB5R-overexpressing flies (CYB5R-OE) and created a transgenic mouse line overexpressing CYB5R3 (CYB5R3-Tg) in the C57BL/6J background to investigate the function of this class of enzymes as regulators of metabolism and age-associated pathologies. Gender- and/or stage-specific induction of CYB5R, and pharmacological activation of CYB5R with tetrahydroindenoindole extended fly lifespan. Increased expression of CYB5R3 was associated with significant improvements in several metabolic parameters that resulted in modest lifespan extension in mice. Diethylnitrosamine-induced liver carcinogenesis was reduced in CYB5R3-Tg mice. Accumulation of high levels of long-chain polyunsaturated fatty acids, improvement in mitochondrial function, decrease in oxidative damage and inhibition of chronic pro-inflammatory pathways occurred in the transgenic animals. These results indicate that CYB5R represents a new target in the study of genes that regulate lipid metabolism and healthspan.

## Introduction

The study of metabolic parameters associated with increased healthspan offers an opportunity to gain new insights into the molecular networks that control and delay age-associated pathologies. Pro-survival properties of antioxidants have been shown in several disease models, although so far only a few interventions with antioxidants have successfully increased lifespan.^1,2^ Calorie restriction (CR) is the most effective non-genetic intervention to delay aging and age-related diseases and to mount effective antioxidant defenses.^3,4^ The value of such health benefits could be dependent to some extent on the CR-mediated induction of cytochrome *b*_*5*_ reductase 3 (CYB5R3),^5,6^ as overexpression of the CYB5R3 ortholog NQR1 increases chronological and replicative lifespan in *Saccharomyces cerevisiae.*^7^

Cytochrome *b*_*5*_ reductases are a family of flavoproteins that catalyze the reduction of coenzyme Q and cytochrome *b*_*5*_ using NADH as an electron donor in a one-electron transfer reaction.^8^ This family is composed of only a single member in *Drosophila melanogaster* (CYB5R) and four members in mammals, known as CYB5R1–4. These proteins exhibit antioxidant properties, as evidenced by an increase in reduced coenzyme Q content coupled with NADH oxidation.^8^ Cytochrome *b*_*5*_ reductases are expressed in a number of subcellular compartments, including the endoplasmic reticulum, the mitochondrial outer membrane and the plasma membrane, to control the redox state of the cells.^5^ CYB5R participates in a variety of metabolic conversions, including the elongation and desaturation of fatty acids, cholesterol biosynthesis and cytochrome P450-mediated mono-oxygenation, and represents a major antioxidant in response to vitamin E deficiency.^9^ The increase in NADH oxidation by CYB5R activity has been linked to the activation of the sirtuin family of NAD^+^-dependent histone deacetylases,^10,11^ and therefore CYB5R may have an important role in the regulation of metabolic pathways associated with healthspan and aging.^12^

To gain new insights into the physiological role of CYB5R in the context of metabolism and aging, we created a transgenic mouse line overexpressing the rat *CYB5R3* gene (CYB5R3-Tg) in the C57BL/6J background and generated *D. melanogaster* overexpressing *Drosophila* CYB5R (CYB5R-OE). Both experimental models were found to live longer than their wild-type counterparts, and reduced tumor burden from *N*-diethylnitrosamine (DEN)-induced liver carcinogenesis was noted in CYB5R3-Tg mice. Additional experiments showed positive effects of CYB5R3 overexpression on mitochondrial function and improved antioxidant defenses with concomitant reduction in oxidative damage. Taken together, these findings have implications for further development of interventions that target CYB5R’s function as regulator of metabolism and age-associated pathologies.

## Results

### CYB5R overexpression extends lifespan, survival and improves lipid metabolism in flies

**Figure 1:**
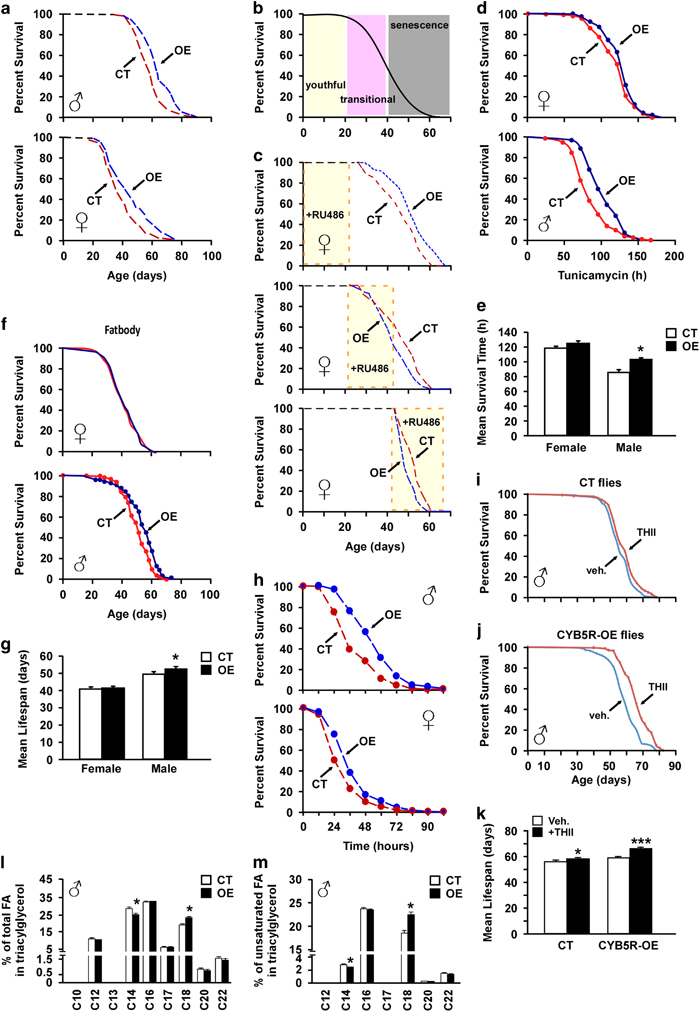
Pleiotropic effects of CYB5R overexpression in flies. (**a**) Kaplan–Meier survival curve for CYB5R-OE and CT flies: males, *n*=120–190 per group (upper panel) and females, *n*=120–220 per group (lower panel). Extension of 20% maximal lifespan in CYB5R-OE flies was significant both in males (*χ*^2^=36.77) and females (*χ*^2^=22.32) at *P*<0.0001. (**b**) Developmental stages in flies. (**c**) Kaplan–Meier survival curve for female flies treated with RU486: young, *n*=120–140 per group (upper panel), transitional, *n*=143–144 per group (middle panel), and old, *n*=89–147 per group (bottom panel). (**d**) Kaplan–Meier survival curve for female (upper panel) and male (lower panel) CYB5R-OE and CT flies treated with vehicle or 12 μm tunicamycin for the next 140 h, *n*=140–150 per group. (**e**) Bars represent mean±s.e.m. of average mean lifespan. *P<0.05. (**f**) Kaplan–Meier survival curve for female (upper panel) and male (lower panel) CYB5R-OE, and CT flies with fat body-specific expression of CYB5R, *n*=160–210 per group. (**g**) Bars represent mean±s.e.m. of average mean lifespan. *P<0.05. (**h**) Kaplan–Meier survival curve for male (upper panel) and female (lower panel) CYB5R-OE, and CT flies treated with paraquat (20 mm in 5% sucrose) for the next 108 h, *n*=120 flies per group. Survival was significantly higher for male (*P*<0.001, *χ*^2^=12.7) and female (*P*=0.04, *χ*^2^=4.2) CYB5R-OE flies. (**i**,**j**) Kaplan–Meier survival curves of male CYB5R-CT (**i**) and CYB5R-OE (**j**) flies treated without and with 100 μm THII. (**k**) Bars represent mean±s.e.m. of male average mean lifespan. *P<0.05, ***P<0.001 compared with control flies. (**l**,**m**) Lipidomic analysis depicting percentages of the different species of total (**l**) and unsaturated (**m**) fatty acyl chains in triacylglycerol in male flies, *n*=5 pools of 8–10 flies per group. Bars represent the mean±s.e.m. *P<0.05.

Flies overexpressing the *CYB5R* gene (CG5946; CYB5R-OE) were created (Supplementary Figure S1A) and validated by showing an approximately twofold increase in CYB5R enzymatic activity in male CYB5R-OE versus control (CT) flies (Supplementary Figure S1B). The log-rank test indicated that male CYB5R-OE flies lived longer with a 10.59% increase in mean lifespan, 10.67% extension in 20% survival (*P*=0.011, *χ*^2^=10.6), and 8.1% increase in 10% survival (*P*=0.0142, *χ*^2^=6; Figure 1a, upper panel). Similarly, female CYB5R-OE flies exhibited a 17.47% increase in mean lifespan, 18.50% extension in 20% survival (*P*=2.27e−05, χ^2^=17.9), whereas a 17.2% increase in 10% survival was observed in CYBR5-OE versus CT flies (*P*=0.0011, *χ*^2^=10.6; Figure 1a, lower panel). Despite sex differences in food consumption, calorie intake was similar between CYB5R-OE and CT flies (Supplementary Figure S1C). Conditional expression of CYB5R was then carried out by applying the chemical RU486 to the diet of different sets of female flies at various developmental stages, which were identified as ‘young/healthy’, ‘transitional’ and ‘senescence’ (Figure 1b). The compound RU486 induced a twofold increase in CYB5R messenger RNA levels versus vehicle-treated CTs (0.92±0.08 vs. 0.47±0.06, *P*=0.004), which translated into significant increase in both mean and maximum lifespan when given to young flies (*P*=0.0008; Figure 1c, upper panel). In contrast, a reduction in longevity was observed when flies in the transitional and senescence stages were treated with RU486 to induce CYB5R (*P*=0.00375, *χ*^2^=8.4 and *P*=0.0413, *χ*^2^=4.2 for transitional and senescence stage, respectively; Figure 1c, middle and bottom panels). The physiological relevance of CYB5R was highlighted by the significant increase in survival of male transgenic flies exposed to tunicamycin, a known ER stress inducer,^13^ when compared with CT flies (*P*=0.0028; Figure 1d,e). Elongation and desaturation of fatty acids have been linked to body fat amount and lifespan extension in *Caenorhabditis elegans*.^14,15^ Here a fat body-specific expression of the *CYB5R* gene was sufficient to increase mean lifespan in male flies (*P*=0.00045; Figure 1f,g). No lifespan extension was observed in female transgenic flies when treated with tunicamycin or when overexpressing a fat body-specific CYB5R (Figure 1d–g).

Under the acute oxidative stress condition induced by paraquat, male and female CYB5R-OE flies exhibited significant increase in survival relative to gender-matched CTs (Figure 1h), indicating that CYB5R overexpression partly promotes longevity through increased oxidative stress resistance in flies. The antioxidant tetrahydroindenoindole (THII) has chemoprotective properties and confers protection against diet-induced inflammatory and oxidative processes, in part, through CYB5R.^16^ Here THII treatment (100 μm) was effective at increasing the average mean lifespan in male CT and CYB5R-OE flies (Figure 1i–k). The log-rank test indicated that there was an 8.95% increase in 20% survival (*P*=0.000629, *χ*^2^=11.7) and a 7.38% increase in 10% survival (*P*=0.016, *χ*^2^=5.8) in CYB5R-OE flies treated with THII versus vehicle. In contrast, THII treatment did not increase lifespan of CT flies (*P*=0.122 for 20% survival and *P*=0.178 for 10% survival).

CYB5R participates in a variety of metabolic conversions, including the desaturation and elongation of fatty acids.^17^ Polyunsaturated fatty acids increase the fluidity of cellular membranes and promote lifespan extension.^18^ Hence, the role of CYB5R in lipid metabolism was examined by assessing the length and degree of saturation of lipid acyl chains. Compared with control flies, both male and female CYB5R-OE flies had reduced levels of relatively short-chain fatty acids, but greater levels of relatively long-chain fatty acids (Figure 1l; Supplementary Figure S1D). Further analysis indicated lower concentrations of unsaturated short-chain fatty acids and higher amounts of longer unsaturated fatty acyl chains in CYB5R-OE males (Figure 1m) and females (Supplementary Figure S1E). Changes in the degree of fatty acid desaturation in male and females flies are depicted in Supplementary Figures S1F,G.

### Lifespan extension and body composition change in CYB5R3-Tg mice

**Figure 2:**
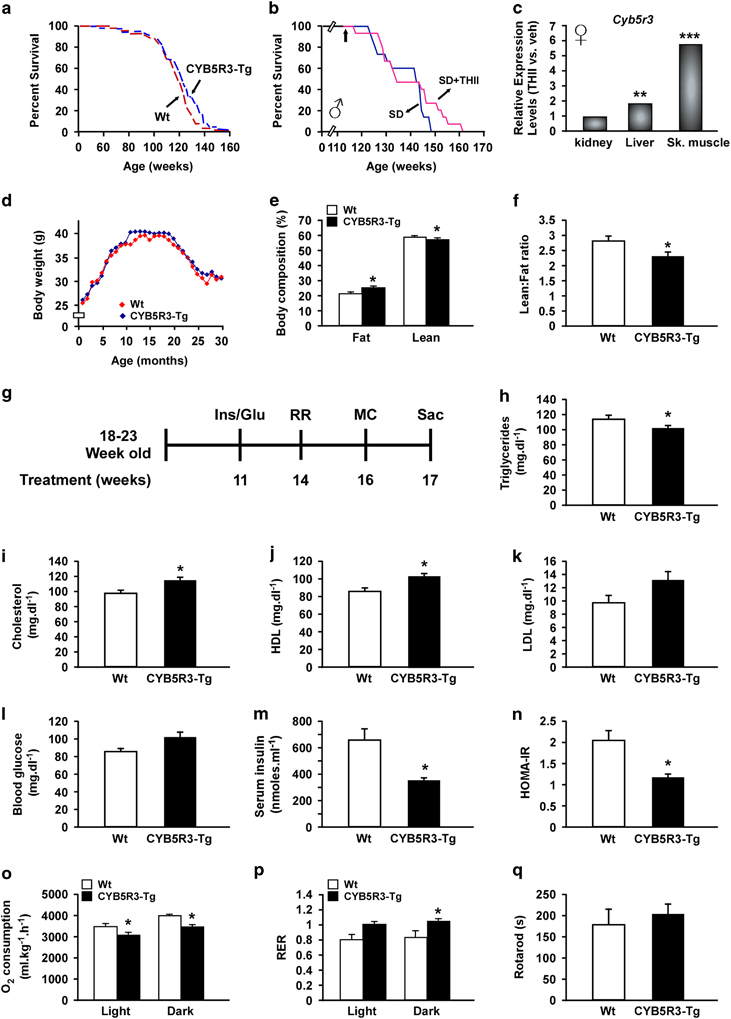
Improved metabolic homeostasis in CYB5R3-Tg mice. (**a**) Kaplan–Meier survival curve for CYB5R3-Tg (*n*=69) and Wt (*n*=64) male mice. Extension of 20% maximal lifespan in CYB5R3-Tg mice was significant (*P*=0.0088, two-tailed *t*-test). (**b**) Kaplan–Meier survival curves of male mice fed SD alone (*n*=14) or supplemented (*n*=16) with 2.7 mg THII per kg body weight. The arrow at 113 weeks indicates the age at which THII treatment was started. (**c**) Relative expression value of *Cyb5r3* transcript assessed by microarray analysis of RNA isolated from the kidney, liver and skeletal muscle of TFII-fed mice versus control animals, *n*=4 per group. **P<0.01 and ***P<0.001. (**d**) Body weight profile over the lifespan. Data include all live animals at each time point. (**e**) Lean and fat percentages in mice were determined by nuclear magnetic resonance (NMR). (**d**,**e**) *n*=64–69 mice per group. (**f**) Lean-to-fat ratio. *n*=6–13 mice per group. (**g**) Protocol design. Male mice (18–23 weeks old) were fed SD (*n*=8 CYB5R3-Tg and *n*=15 Wt mice) or HFD (Supplementary Section) for 17 weeks. At the indicated time points (weeks), the following measures were performed: Ins/Glu, insulin and glucose determination; RR, rotarod; MC, metabolic cages; Sac, sacrifice of the animals. (**h**–**n**) The following analyses were carried out in mice after a 20-h fasting period: (**h**) serum triglycerides; (**i**) total serum cholesterol; (**j**) high-density lipoprotein (HDL) content; (**k**) low-density lipoprotein (LDL) content; (**l**) blood glucose levels; (**m**) serum insulin levels; and (**n**) HOMA-IR index. (**h**–**k**) *n*=5 per group; (**l**–**n**) *n*=7–12 per group. (**o**,**p**) Sixteen weeks after SD feeding, mice were placed into metabolic cages for the measure of (**o**) *in vivo* oxygen consumption and (**p**) respiratory exchange ratio (RER), *n*=4–6 per group. (**q**) Time to fall from an accelerating rotarod, *n*=7–15 per group. Data are represented as the mean±s.e.m. *P<0.05 compared with Wt mice.

A transgenic mouse strain overexpressing the rat *CYB5R3* gene was created (CYB5R3-Tg) in the C57BL/6J background and validated (Supplementary Figure S2A–D). When maintained on a standard diet (SD), CYB5R3-Tg male mice displayed an extended medium and maximum longevity (Figure 2a). The survival curve of CYB5R3-Tg started to diverge from Wt mice (*n*=64–69 per group) at 112 weeks of age and progressively deviated until the end of the study, with a modest 3.08% increase in mean lifespan (*P*=0.04, *χ*^2^=4.09 in a log-rank test). In CYB5R3-Tg mice, the 6.54% increase in 20% survival was significant (*P*=0.0088 in a two-tailed *t*-test), whereas the 5.89% increase in 10% survival did not reach statistical significance (*P*=0.0603 in a two-tailed *t*-test). No obvious differences in the cause of death between Wt and CYB5R3-Tg mice were noted. Necropsy and gross examination indicated age-associated hyperplasia of several tissues and common neoplasms (liver and spleen), which are the likeliest causes of death in these animals (Supplementary Table S1). The log-rank test indicated that THII treatment of 113-week-old male mice on SD (*n*=14–16 per group) did not extend significantly the lifespan of mice when started at 2 years of age (*P*=0.081, *χ*^2^=3.044; Figure 2b) nor did the body weight trajectories (data not shown). A 10-week administration of THII led to a significant 1.91- and 5.77-fold induction in *Cyb5r3* transcript in liver and muscle, respectively (Figure 2c).

CYB5R3-Tg mice had similar body weight as Wt animals when fed SD in the longevity study (Figure 2d) or when measured in a different cohort of mice fed either high-fat diet (HFD) or SD (Supplementary Figure S2E). Calorie intake did not differ between Wt and CYB5R3-Tg mice maintained on both diets, even when corrected for body weight (Supplementary Figure S2F,G). CYB5R3-Tg mice exhibited lower lean-to-fat ratio due to their higher percentage body fat (Figure 2e,f). These results indicate that CYB5R3 overexpression can extend lifespan in mice by mechanisms that may be distinct from those described for CR.

### Energy balance and physical fitness of CYB5R3-Tg mice

The regulation of glucose homeostasis, energy balance and physical fitness was investigated in CYB5R3-Tg mice starting at the age of 18–23 weeks and fed with SD or HFD for the next 17 weeks (see study design in Figure 2g). Eleven weeks into the SD diet, fasted CYB5R3-Tg mice had lower circulating triglyceride concentrations and higher total cholesterol levels associated with the high-density lipoprotein fraction (Figure 2h–j), whereas a non-significant trend towards higher low-density lipoprotein levels was observed with CYB5R3 overexpression (Figure 2k). Fasting glucose levels were within normal range (86.33±3.48 vs. 101.85±6.55 mg dl^−1^ in Wt versus CYB5R3-Tg, respectively, *P*=0.064; Figure 2l), whereas circulating insulin was significantly lower in fasted CYB5R3-Tg mice (Figure 2m), which translated into a significantly lower HOMA-IR index in CYB5R3-Tg versus Wt mice (Figure 2n). Like with SD feeding, HFD-fed CYB5R3-Tg mice displayed increased insulin sensitivity and improved glucoregulation (Supplementary Figure S2H–J).

Sixteen weeks into the SD diet, mice were placed into separate metabolic chambers to provide estimates of energy expenditure during fasting and fed periods. As anticipated, CYB5R3-Tg mice, which are fatter than Wt controls (see above), consumed less oxygen per gram of body weight (Figure 2o) consistent with the well-established fact that fat is less metabolically active than lean tissues. The marked increase in respiratory exchange ratio in CYB5R3-Tg mice indicated preferential use of carbohydrates to meet their energy needs (Figure 2p). Spontaneous locomotion was similar in the two groups of mice on SD (Supplementary Figure S2K), and the overall endurance, as assessed by the rotarod test, showed no difference whether mice were on SD (Figure 2q) or HFD (Supplementary Figure S2L). Measures of the core body and interscapular temperatures also showed no significant differences between Wt and CYB5R3-Tg mice that had been fed, fasted or administered glucose by an oral glucose gavage (1.5 g kg^−1^ body weight; Supplementary Figure S2M–O). Altogether, these results indicate that the lifespan extension in CYB5R3-Tg mice correlated with improvement in the control of lipid and glucose homeostasis.

### Liver microarray and protection against inflammation, oxidative stress and cancer

**Figure 3:**
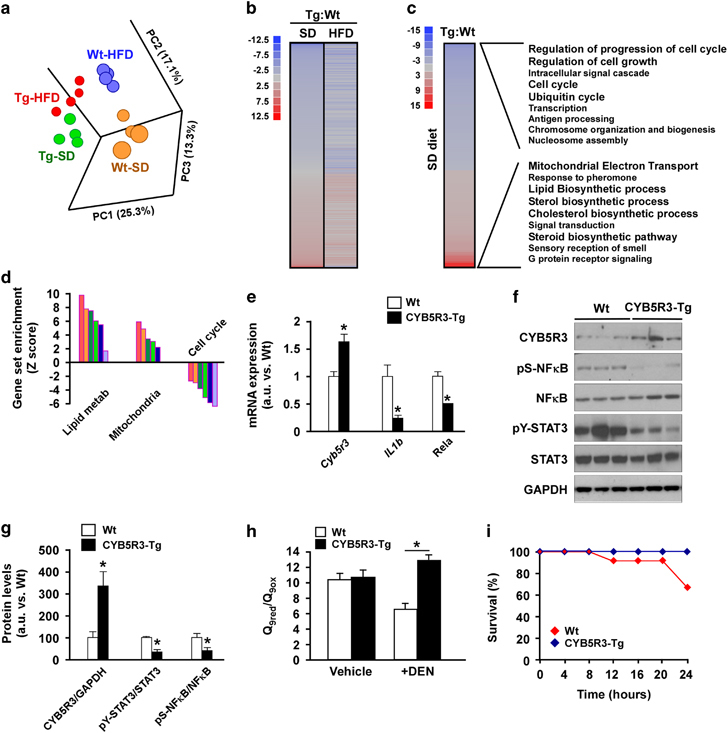
Overexpression of CYB5R3 leads to alterations in inflammatory pathways and redox cycle in mouse liver. (**a**) Principal component analysis (PCA) from microarray RNA experiment in livers of 11-month-old CYB5R3-Tg mice maintained on either SD or HFD compared with their respective Wt controls. (**b**) Heat maps comparing liver transcripts significantly upregulated (red) and downregulated (blue) by CYB5R3 overexpression in mice fed SD and HFD diets. (**c**) Heat maps depicting gene sets significantly upregulated (red) and downregulated (blue) in SD-fed CYB5R3-Tg versus Wt mice. (**d**) Effect of CYB5R3 overexpression on lipid metabolism (lipid metab), mitochondria and cell cycle gene sets in mice fed SD. For microarray analysis, *n*=4 per group. (**e**) Hepatic IL1-β and RELA messenger RNA (mRNA) levels in 11-month-old CYB5R3-Tg mice by quantitative reverse transcription PCR, *n*=3–4 per group. Values were normalized to Wt controls. (**f**) Western blots indicating activation of pro-inflammatory signaling pathways in the liver of 11-month-old CYB5R3-Tg mice. (**g**) Densitometric analysis of western blots in **f**, *n*=3–7 per group. (**h**) Ratio of reduced-to-oxidized coenzyme Q_9_ in the liver of 5-month-old mice treated with DEN (100 mg kg^−1^ body weight, intraperitoneal injection) or vehicle for 48 h, *n*=4–5 per group. (**i**) Mice (18–25 months old) were treated with diquat (60 mg kg^−1^ body weight, intraperitoneal injection) and survival was monitored for the next 24 h, *n*=12 per group. Data are represented as the mean±s.e.m. *P<0.05 compared with Wt or control mice.

Principal component analysis revealed a clear effect of CYB5R3 overexpression and diet on the liver transcriptome profiles (Figure 3a). Heat map analysis showed extensive differential transcript expression between CYB5R3 and Wt livers, irrespective of the diet (Figure 3b). Polo-like kinase 3 (*Plk3*), which encodes a cytokine-inducible protein that participates in the regulation of cell cycle progression and tumorigenesis, was the top downregulated transcript in the liver of SD-fed CYB5R3-Tg mice. Parametric analysis of gene set enrichment revealed a perturbation of several functional pathways in response to CYB5R3 overexpression (Figure 3c). Lipid biosynthesis, mitochondrial function and cell cycle control were among the ~200 gene sets significantly impacted in the CYB5R3-Tg:Wt pairwise comparison (Figure 3d; Supplementary Table S2). Of significance, there was robust CYB5R3-inducible expression of transcripts associated with the responses to xenobiotics and inflammation (e.g., cytochrome P450 proteins) and aerobic respiratory pathways (e.g., coenzyme Q biosynthesis and oxidative phosphorylation (Supplementary Figure S3,4). The pro-inflammatory cytokine interleukin 1β (*Il1b*) and the NF-κB p65 subunit (*Rela)* were among the messenger RNA transcripts significantly downregulated in CYB5R3-Tg versus Wt livers (Figure 3e), as were the levels of phospho-active forms of NFκB (pNFκB) and STAT3 (pSTAT3) (Figure 3f,g).

By contributing to the maintenance of coenzyme Q in its reduced form in cellular membranes, CYB5R3 helps protect against endogenous and exogenous oxidative damage and promotes aerobic metabolism.^7^ Here CYB5R3 overexpression conferred significant protection of the hepatic coenzyme Q_9_ redox state after a 48-h treatment with the pro-oxidant xenobiotic DEN in mice (Figure 3h). Similarly, 24 h after administration of diquat, a potent pro-inflammatory and oxidative stress agent,^19^ more than ~40% death was observed in Wt and none in CYB5R3-Tg mice (Figure 3i*; P*<0.01, *χ*^2^=5.99). These findings suggest that CYB5R3 overexpression promotes longevity, at least partially, through increased oxidative stress resistance.

**Figure 4:**
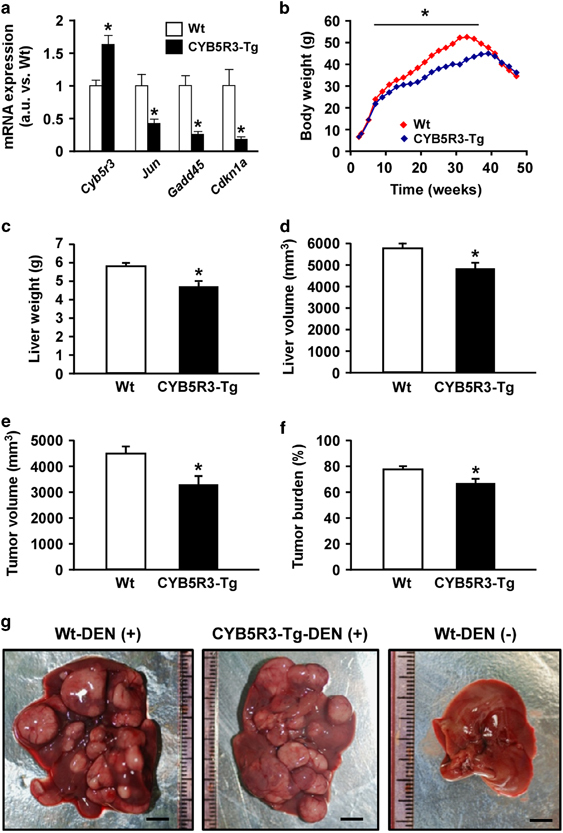
CYB5R3 overexpression protects against chemically induced liver cancer. (**a**) Determination of messenger RNA (mRNA) levels of several hepatic cell cycle regulatory genes in 11-month-old mice fed SD, *n*=3–4 per group. Values were normalized to Wt controls. (**b**) Body weight during DEN protocol. Data includes all live animals in each time point. (**c**) Liver weight at sacrifice, *n*=5–11 per group. (**d**–**f**) Magnetic resonance imaging analysis was carried out at sacrifice, *n*=4–10 per group: (**d**) liver volume; (**e**) tumor volume; and (**f**) tumor volume ratio, e.g., percent of tumor volume divided by liver volume. (**g**) Representative photographs of livers from CYB5R3-Tg and Wt mice included in the DEN protocol. Bar, 5 mm. Data are represented as the mean±s.e.m. *P<0.05 compared with Wt mice.

A decline in expression of a number of messenger RNAs involved in cell cycle progression was observed in CYB5R3 livers (Figure 4a), which led us to ascertain whether CYB5R3 overexpression confers protection against cancer development. Sixteen-day-old mice were injected with DEN, a carcinogenic agent that promotes G1/S-phase regulatory protein induction,^20^ and, after weaning, were put on HFD for 11 months. This approach, which enables the development of metabolic syndrome-associated liver cancer in mice,^21^ caused significant differences in weight trajectories between CYB5R3-Tg and Wt mice (Figure 4b). In response to DEN, 7-week-old CYB5R3-Tg mice weighted less than age-matched Wt animals; however, body weights of Wt mice at 35 weeks of age started to drop precipitously, whereas CYB5R3-Tg mice maintained their body weights until week 41 (Figure 4b). At 10 months of age and until the end of the study, CYB5R3-Tg mice tended to have higher average body weight than Wt animals. At the time of the sacrifice, liver weights were 19.5±5.7% (*P*=0.01) smaller in DEN-treated CYB5R3-Tg compared with Wt mice (Figure 4c). Postmortem magnetic resonance imaging analysis showed a total liver volume of 4,820±285 and 5,742±213 mm^3^ in DEN-treated CYB5R3-Tg and Wt mice, respectively, which translated to a 17.1±5.4% (*P*=0.02) reduction in the transgenic animals (Figure 4d). There was a 27.2±7.8% (*P*=0.02) and 16.6±5.2% (*P*=0.03) reduction in cancer volume and percentage of tumor volume (compared with the total liver volume), respectively, in DEN-treated CYB5R3-Tg versus Wt mice (Figure 4e,f; Supplementary Figure S5). Differences in size between Wt and CYB5R3-Tg livers were apparent during necropsy (Figure 4g).

### Effect of CYB5R3 overexpression on hepatic fatty acyl chain desaturation and elongation

**Figure 5:**
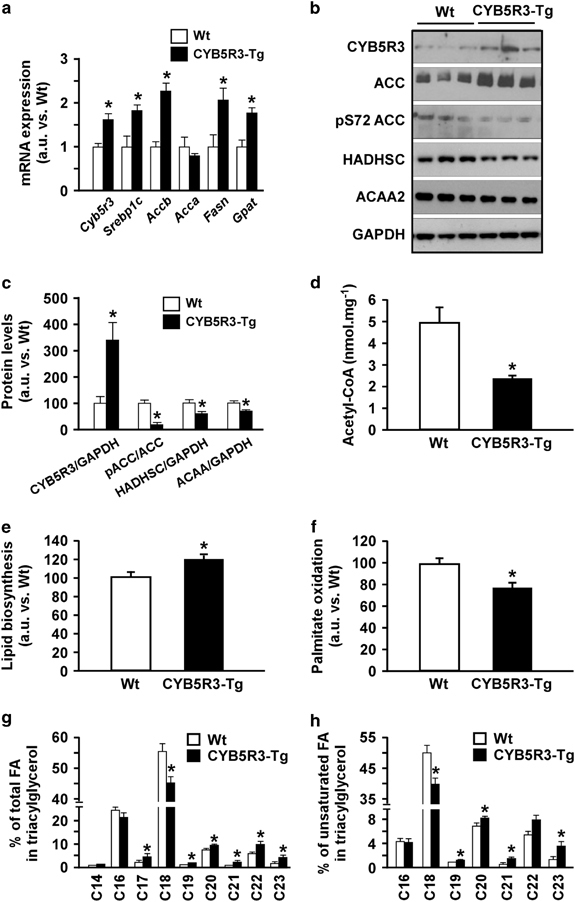
Regulation of hepatic lipid metabolism in CYB5R3-Tg mice. (**a**) Messenger RNA (mRNA) levels of hepatic genes involved in lipid biosynthesis in 11-month-old mice fed SD, *n*=3–4 mice per group. Values were normalized to Wt controls. (**b**) Western blots showing higher acetyl-CoA carboxylase (ACC) activation in CYB5R3-Tg mice and lower abundance of proteins involved in mitochondrial fatty acid β-oxidation, *n*=3–7 per group. (**c**) Densitometric analysis of western blots depicted in **b**. (**d**) Total liver acetyl-CoA levels, *n*=7 per group. (**e**) Glucose incorporation into lipids in isolated primary hepatocytes of 3-month-old mice fed SD, *n*=7 per group. (**f**) Palmitate oxidation in isolated primary hepatocytes of 3-month-old mice fed SD, *n*=9 per group. (**g**,**h**) Lipidomic analysis depicting percentages of the different species of total (**g**) and unsaturated (**h**) fatty acyl chains in triacylglycerol in the livers of 5-month-old CYB5R3-Tg and Wt mice fed SD, *n*=4 per group. FA, fatty acids. Data are represented as the mean±s.e.m. *P<0.05 compared with Wt animals.

Several transcripts encoding enzymes required for the synthesis of various lipid molecular species were induced in CYB5R3-Tg livers (Figure 3d; Supplementary Table S2; Supplementary Figure S3), which is consistent with the regulatory function of CYB5R3 in fatty acid chain elongation and desaturation. Expression of hepatic genes involved in fatty acid synthesis (Figure 5a) greater amount of the lipogenic enzyme acetyl coenzyme A carboxylase was induced by CYB5R3 overexpression together with concomitant reduction in the levels of hydroxyacyl coenzyme A dehydrogenase and acetyl coenzyme A acyltransferase, two mitochondrial fatty acid β-oxidation enzymes (Figure 5b,c). The accumulation of a dephosphorylated, ‘active’ pool of acetyl coenzyme A carboxylase^22^ (Figure 5b,c) coincided with significant reduction in hepatic acetyl-CoA levels in CYB5R3-Tg mice (Figure 5d). Last, CYB5R3 overexpression was effective in promoting a substantial 18.4±5.0% increase in *de novo* lipogenesis (*P*=0.02) with a concomitant 24.2±5.4% reduction in lipid β-oxidation (*P*=0.007) in primary culture of adult mouse hepatocytes (Figure 5e,f).

Unlike hepatic cholesterol level that was identical in both groups of mice (44±2 vs. 42±3 nmol mg^−1^ protein, *n*=5 per group), the length and degree of saturation of lipid acyl chains in triglycerides was influenced by CYB5R3 overexpression. Shotgun lipidomics revealed a higher proportion of very long-chain fatty acids (C19–C23) but lower amount of C18 fatty acyl chains in CYB5R3-Tg versus Wt livers (Figure 5g; Supplementary Figure S6). Moreover, the levels of unsaturated short-chain fatty acids declined, whereas those of longer unsaturated fatty acyl chains increased (Figure 5h; Supplementary Figure S6), consistent with hepatic fatty acyl chain elongation and desaturation in CYB5R3-Tg mice. This follows a pattern similar to that of CYB5R-OE flies (Figure 1l; Supplementary Figure S1D–G). Noteworthily, the lipid composition in AIN-93G chow is based on soybean oil, a rich source of C16 and C18 fatty acids devoid of fatty acids beyond 18 carbons (Supplementary Tables S3 and S4).

### Mitochondrial efficiency is improved in CYB5R3-Tg livers

**Figure 6:**
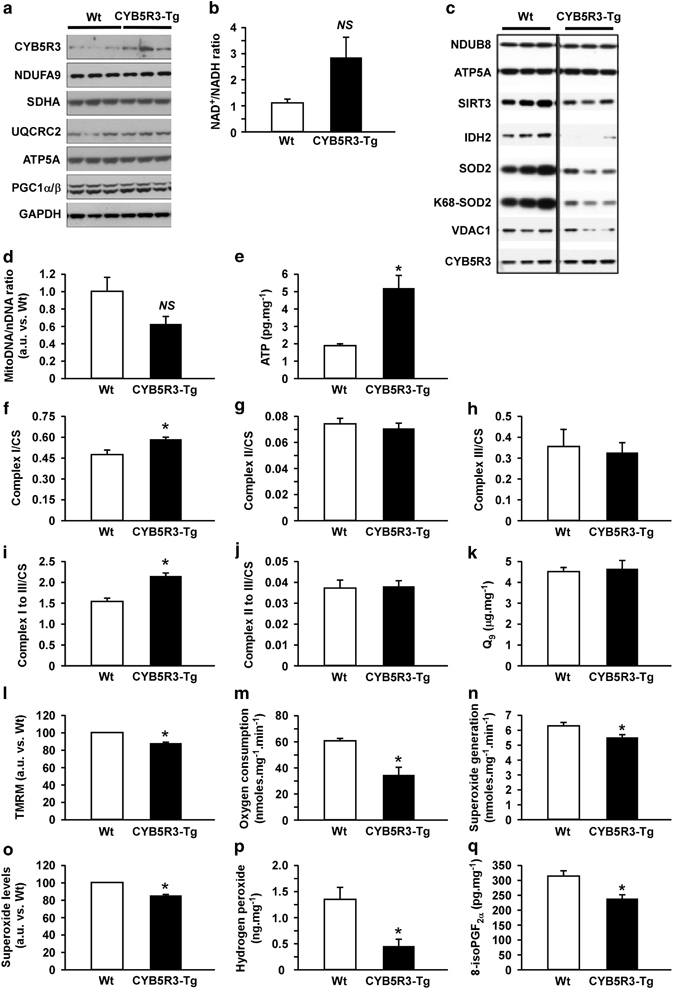
Energy homeostasis and antioxidant protection in CYB5R3-Tg mice. (**a**) Hepatic expression in mitochondrial proteins, *n*=3–7 mice per group. (**b**) NAD^+^-to-NADH ratio, *n*=7–8 per group. (**c**) Mitochondrial fractions were prepared and immunoblotted against the indicated primary antibodies, *n*=5–7 mice per group. (**d**) Mitochondrial to nuclear DNA ratio was measured by quantitative reverse transcription PCR, *n*=7–8 per group. (**e**) Hepatic ATP content, *n*=5–6 per group. (**f**–**j**) Mitochondrial electron transport chain (ETC) activities: (**f**) complex I, (**g**) complex II, (**h**) complex III, (**i**) complex I– III and (**j**) complex II–III. Data were normalized to citrate synthase (CS) activity, *n*=6–7 per group. (**k**) Coenzyme Q_9_ levels in livers of 5-month-old mice, *n*=8 per group. (**l**) Mitochondrial membrane potential and (**m**) efficiency, as defined as the minimal oxygen consumption under state III respiration conditions, were measured in freshly isolated liver mitochondria, *n*=3 trials per group. (**n**) Hepatic superoxide production, *n*=6–7 per group. (**o**) Superoxide levels determined by Mitosox staining in freshly isolated mitochondria fraction, *n*=3 per group. (**p**) Hydrogen peroxide levels, *n*=5–8 per group. (**q**) Lipid peroxidation determined as the accumulation of 8-iso-PGF_2α_, *n*=6–7 per group. Unless otherwise indicated, all analyses were performed in liver extracts or mitochondria of 11-month-old male CYB5R3-Tg and Wt mice fed SD. Data are represented as the mean±s.e.m. *P<0.05 versus Wt mice.

The maintenance of efficient mitochondrial performance has been associated with lower accumulation of oxidative stress, coupled with increased healthspan and longevity.^23^ Unlike the expression of several subunits of the electron transport chain complexes and PGC1-α/β, a master regulator of mitochondrial biogenesis, (Figure 6a), there was a trend towards increased NAD^+^/NADH ratio (Figure 6b; *P*=0.07) and clear reduction in SIRT3 levels in CYB5R3-Tg livers (Figure 6c). The mitochondrial-to-nuclear DNA ratio was not affected by CYB5R3 overexpression (Figure 6d, *P*=0.15) despite the ~2.5-fold increase in ATP levels (Figure 6e). The upregulation in complex I and I–III activities, but not in complex II, complex II–III, complex III activities or in coenzyme Q_9_ levels (Figure 6f–k) was consistent with a preferential use of carbohydrates in CYB5R3-Tg mice. CYB5R3 overexpression was associated with lower membrane potential with higher mitochondrial efficiency in freshly isolated liver mitochondria (Figure 6l,m). Superoxide production was reduced in CYB5R3-Tg livers (Figure 6n) and freshly isolated preparations of CYB5R3-Tg liver mitochondria subjected to Mitosox staining also showed lower superoxide levels (Figure 6o). The mitochondrial content of the antioxidant proteins SOD2 and IDH2 was markedly lower in CYB5R3-Tg livers (Figure 6c), and paralleled the significant reduction in the hepatic levels of hydrogen peroxide and 8-isoPGF_2α_, a marker of lipid peroxidation (Figure 6p,q).

## Discussion

Our data, generated from new fly and mouse models of CYB5R/CYB5R3 overexpression/transgenesis, support the notion that healthier lifespan can be achieved through increased expression and/or activity of this NADH-dependent oxidoreductase. Microarray data coupled with gene set enrichment analysis indicates that ‘regulation of progression of cell cycle’ and ‘regulation of cell growth’ are the most downregulated pathways in CYB5R3-Tg liver. Therefore, the significant increase in 20% survival of CYB5R3-Tg mice implies that the end-of-life pathology data were collected later in the transgenic animals than their Wt counterparts. We posit that CYB5R3 overexpression slows age-associated tumor progression rather than conferring cancer protection, as the occurrence of spontaneous cancer at the end of life did not differ between the two genotypes. These observations suggest that lifespan extension could be achieved by delaying the onset of tumorigenesis.

It is gradually being recognized that interventions that extend lifespan are associated with lower accumulation of oxidative damage.^24^ Mice deficient in NCB5OR, a second member of the CYB5R family, exhibit a diabetic phenotype due to significantly greater fatty acid oxidation rates and palmitate-induced oxidative stress responses in hepatocytes,^25–27^ which is consistent with the idea that chronic inflammation leads to the development of metabolic complications through the activation of transcription factors such as NF-κB and STAT3, and subsequent increase in expression of inflammation-related genes.^28,29^ CR induces plasma membrane CYB5R3 to support antioxidants recycling and prevent fatty acid peroxidation and ceramide-dependent apoptosis.^5,8^ It has been proposed that transient knockdown of CYB5R3 leads to a premature senescence-like phenotype in human MRC-5 cells and in human fibroblasts harboring missense mutations in this gene.^12^ Furthermore, CYB5R3 helps maintain genomic stability by promoting detoxification of xenobiotics by mono-oxygenation of P450 proteins.^30^ In line with the concepts that accumulation of oxidative damage and lower mitochondrial functionality are contributory factors to the development of most age-related diseases, our observations in flies and mice overexpressing CYB5R provide direct evidence that interventions aimed at promoting antioxidant defenses can increase healthspan, delay age-related metabolic dysfunctions and modestly extend longevity.

Translating the beneficial effects on healthspan between CR and CYB5R3 overexpression is difficult but ideally should take into account a comparison of the biosynthetic and bioenergetic pathways. CR reduces the age-related accumulation of long-chain unsaturated fatty acids (UFA),^31^ and studies using CR or metformin reveal that increased longevity requires the use of fat as a source of fuel.^32^ Here a positive association between long-chain UFA levels and antioxidant protection both in CYB5R3-Tg mice and CYB5R-OE flies was found, consistent with greater degree of membrane fluidity^33^ and improved health.^34^ When placed in metabolic chambers, the transgenic mice showed preference for carbohydrate consumption over utilization of fatty acid oxidation for their energy needs. In addition, the increase in mitochondrial complex I activity in the liver of CYB5R3-Tg mice was accompanied by enhanced lipogenesis both *in vivo* and *in vitro*, and lower expression of genes encoding proteins implicated in mitochondrial β-oxidation. These data are at variance with the mechanisms used by CR to extend lifespan. CYB5R3-Tg mice required also less insulin to metabolize glucose and generate ATP, suggestive of a shift in mitochondrial respiration efficiency. We believe that the improvement in insulin sensitivity in the transgenic animals stem from the marked reduction in pro-inflammatory signaling combined with an increase in hepatic long-chain UFA levels, which could preserve membrane fluidity and signal transduction. Notable is the study by Kunau *et al.*^35^ in which saturated fatty acids were found to enable mitochondrial β-oxidation to proceed. This report leads us to propose that high UFA levels hamper β-oxidation of fatty acids in CYB5R3-Tg liver. Further investigation is warranted to better understand the contribution of metabolism in driving the aging process.

With glucose as metabolic fuel, coenzyme Q-driven flow of electrons from complex I to complex III is preferred over that of complex II to complex III.^36^ Our observation that CYB5R3-Tg mice have greater use of carbohydrates suggests that electrons are carried to the electron transport chain through complex I at the expense of complex II utilization. An increase in mitochondrial efficiency, defined as the minimal oxygen consumption under state III conditions, was observed in CYB5R3-Tg liver together with lower mitochondrial membrane potential, reduced generation and accumulation of oxidative damage, and higher ATP levels. CYB5R3 associates with the outer membrane of mitochondria and reacts with various redox partners,^12,37^ thus enabling the reduction of endogenous cytochrome *c* through transfer of electrons downstream of the reactive oxygen species (ROS)-producing mitochondrial complexes I and II.^38^ Although low production of a diffusible agent, such as superoxide, is channeled to cytochrome *c* and used to potentiate mitochondrial efficiency, higher amounts may not be detoxified and would most likely produce damage, which is suggestive of hormesis.^39^ CYB5R3 has the ability to act as a NADH-dependent source of superoxide anion^40^ through the production of semi-ubiquinone radical.^41^ It is plausible that CYB5R3 overexpression might increase mitochondrial efficiency to produce more ATP, which, in turn, could lower accumulation of oxidative damage and improve healthspan. Expression of the *C. elegans* Fat1 desaturase in mice, which also increases fatty acid desaturation and decreases ROS production by complex I,^42^ lend further credence to our results.

Highly unsaturated fatty acids have a high peroxidability index, which is determined by the number of carbon double bonds that can be oxidized.^43^ The high melting point of peroxidized lipids increases membrane rigidity,^44^ thereby altering intracellular signaling pathways and associated cellular processes.^45^ Our observations that a significant reduction in lipid peroxidation occurred despite high levels of UFA in CYB5R3-Tg liver reinforce the notion that CYB5R3 overexpression prevents the accumulation of biomarkers of oxidative stress. Enhanced metabolism of xenobiotics by cytochrome P450 proteins may partly explain the cytoprotection against diquat (Figure 3i) and DEN-induced hepatocarcinoma in CYB5R3-Tg mice (Figure 4b–g). The chemical carcinogen DEN affects preferentially liver tissue where it is converted by P450-dependent monooxygenases into a highly reactive molecule (ethyl radical), with subsequent ROS production and generation of DNA adducts.^46^ Paraquat mediates redox cycling reactions coupled with impairment of the mitochondrial electron transport, which ultimately leads to ROS generation and alterations in cellular bioenergetics.^47^ The increase in paraquat resistance can be achieved through marked reduction in redox cycling necessary for the generation of superoxide anion. NAD(P)H is the main source of reducing equivalents for the intracellular reduction of paraquat,^48^ and potential decreases in cellular NAD(P)^+^/NAD(P)H ratio driven by overexpression of CYB5R3 in the outer mitochondrial membrane may be protective against cycling oxidizing agents.^38^ These findings combined with the fact that DEN, diquat and paraquat have the capacity to generate ROS make it clear that the specific increase in mitochondrial complex I activity and the maintenance of the reduced pool of coenzyme Q9 in response to CYB5R3 overexpression would provide protection against pro-oxidant xenobiotic poisoning. CYB5R3 attenuates the tumor-promoting effects of DEN by delaying tumor growth, rather than inducing global suppression of tumorigenesis.

In summary, this cross-species study along with our previous report in yeast indicate that CYB5R is an important and conserved modulator of healthspan-associated pathways through improved control of energy homeostasis and mitochondrial bioenergetic efficiency. We demonstrated that CYB5R3 overexpression also confers partial protection against xenobiotic-induced liver cancer in laboratory mice. Our findings warrant the development of new strategies aimed at targeting CYB5R for metabolism regulation and treatment of age-associated pathologies.

## Materials and methods

Full details are provided in the Supplementary Information section.

### Transgenesis, animal models and diets

The rat *CYB5R3* gene was cloned into the pRC/CMV-rDTD plasmid.^49^ The transgene insert was cleaved from the DNA cloning vector by digestion with *Swa*I and *Nru*I restriction enzymes. The purified transgene was microinjected into fertilized C57BL/6J eggs at the University of Michigan Transgenic Animal Model Core Facility (http://www.med.umich.edu/tamc/). Surviving eggs were transferred to pseudopregnant B6D2F1 female mice. The construct was stably incorporated into the genome, and expression of the gene was under the control of the human cytomegalovirus immediate-early promoter and the SV40 polyadenylation sequences.

For flies, the UAS-CYB5R fly line was generated by injecting *w*^1118^ fly with pUAST vectors containing full-length CYB5R complementary DNA. CYB5R overexpression under the control of the UAS promoter was induced by da-Gal4, a ubiquitously expressed Gal4 driver. Both da-Gal4 and UAS-CYB5R lines were backcrossed with wild-type *w*^1118^ flies for more than five generations to minimize variations in genetic background before being used for any assay. To determine the effect of CYB5R overexpression on lifespan, UAS-CYB5R males were mated with da-gal4/+ virgin females and the UAS-CYB5R/da-Gal4 progeny were collected as CYB5R-OE flies and UAS-CYB5R/+ from the same cross were collected as control flies. Flies were subsequently cultured on 2.5% sugar–2.5% yeast–1.5% agar diet and transferred to fresh food once every 2–3 days for lifespan assays.

Animal procedures, housing and diets were in accordance with the guidelines issued by the Intramural Research Program of the National Institutes of Health and are described in the Supplementary Informtion.

### Rotarod test

Results from rotarod tests are presented as the time to fall from an accelerating rotarod (4–40 r.p.m. over 5 min). A detailed explanation of the protocol is described in the Supplementary Informtion.

### Glucose and insulin determination

To determine glucose and insulin levels, blood samples were collected by venipuncture and analyzed as described in the Supplementary Informtion.

### Shotgun lipidomics analysis of fatty acyl composition of triacylglycerol

Lipid samples were prepared from mouse liver tissue powders by a modified Bligh and Dyer method as previously described.^50^ Internal standards for quantification of individual lipid molecular species were added before lipid extraction.^50^ Shotgun lipidomics analyses were performed with a QqQ mass spectrometer (Thermo Fisher Scientific TSQ Vantage, San Jose, CA, USA) equipped with an automated nanospray device (TriversaNanomate, Advion Biosciences, Ithaca, NY, USA) and operated with the Xcalibur software (Thermo Fisher Scientific, Inc., Waltham, MA, USA) as previously described.^51^ Identification and quantification of triacylglycerol molecular species were performed as previously described.^52,53^ Analysis of fatty acids was also carried out in 14-day-old male and female flies by gas chromatography (Microbial ID, Inc., Newark, DE, USA).

### DEN-induced hepatocellular carcinoma model

Sixteen-day-old mice were injected intraperitoneally with DEN at 5 mg kg^−1^ of body weight and, at 21 days of age, they were fed a HFD for 11 months. Five Wt and eleven CYB5R3-Tg mice were included in the study. One mouse of each genotype died during the duration of the study, with the Wt animal dying with an enlarged spleen and liver tumors, whereas the CYB5R3-Tg mouse that died had no tumor present, making the cause of death of this animal uncertain. At eleven months, mice were killed and magnetic resonance imaging analysis was performed as described in the Supplementary Informtion. Liver and tumor volumes were estimated from the MR images by counting the pixels in each category using ImageJ 1.42 (National Institutes of Health, Bethesda, MD, USA). Image analysis was performed by at least two researchers blinded to the experimental groups, and similar results were obtained.

### Microarray analysis, quantitative real-time PCR and western blotting

Details can be found in the Supplementary Informtion.

### Statistical analysis

Unless otherwise stated, statistical comparisons between genotypes were performed using paired Student’s *t*-tests. Analyses were performed using Excel 2010 (Microsoft, Redmond, WA, USA). LogRank statistical analyses were performed using SigmaStat 3.5 (Systat Software, San Jose, CA, USA). *P-*values ⩽0.05 were considered significant. Densitometry analyses were performed with ImageJ software (NIH, Bethesda, MD, USA).
