# Supplementary material for: Cytochrome b5 reductase and the control of lipid metabolism and healthspan
Source: NPJ Aging Mech Dis. 2016 May 12;2:16006–. doi: 10.1038/npjamd.2016.6 (PMC5515006; doi:10.1038/npjamd.2016.6)
Supplement: Supplementary Information [file npjamd20166-s1.doc]

**SUPPLEMENTARY INFORMATION**

**Cytochrome *b*_5_ reductase and the control of lipid metabolism and healthspan**

Alejandro Martin-Montalvo, Yaning Sun, Alberto Diaz-Ruiz, Ahmed Ali, Vincent Gutierrez, Hector H. Palacios, Jessica Curtis, Emilio Siendones, Julia Ariza, Gelareh A. Abulwerdi, Xiaoping Sun, Annie X. Wang, Kevin J. Pearson, Kenneth W. Fishbein, Richard G. Spencer, Miao Wang, Xianlin Han, Morten Scheibye-Knudsen, Joe A. Baur, Howard G. Shertzer, Placido Navas, Jose Manuel Villalba, Sige Zou, Michel Bernier, and Rafael de Cabo.

**This section contains:**

• Six Supplementary Figures and their legends ……………………………..… pages 2-z

• Five Supplementary Tables ………………………………………………….. pages x-c

• Supplementary Materials and Methods ……………………………………… pages v-b

• Supplementary References …………………………………………………… pages b-n

**SUPPLEMENTARY FIGURE LEGENDS**

**Figure S1, Related to Figure 1. Characteristics of CYB5R-overexpressing flies**.

(A) CYB5R mRNA levels in CYB5R-OE and control (CT) flies were determined by quantitative RT-PCR. Male flies, n=3-4 pools of 20 heads per group; female flies, n=5 pools of 20 heads per group. (B) CYB5R enzymatic activity in male flies, n=5 pools of 20 flies per group. (C) Food intake of CYB5R-OE male and female flies, respectively; n=16 flies per group. (D-G) Lipidomic analysis depicting percentages of the different species of total (D) and unsaturated (E) fatty acyl (FA) chains in triacylglycerol in female CYB5R-OE and CT flies. Percent of the indicated fatty acid species in male (F) and female (G) flies, n=5 pools of 8-10 flies per group. FA, fatty acids

**Figure S2, Related to Figures 2. Metabolic and behavioral responses in CYB5R3-Tg mice.**

(A) Representative Southern blot analysis of CYB5R3-Tg mice. Genomic DNA from CYB5R3-Tg and wild-type (Wt) mice were digested with EcoRV, blotted and hybridized with a labeled DNA probe. DNA markers are shown on the left (Mr). (B) *Cyb5r3* mRNA levels from multiple tissues of 3-month-old male mice were determined by quantitative RT-PCR. (C) Western blot analysis showed an upregulation in CYB5R3 protein levels in several tissues as compared to Wt animals. (D) CYB5R3 enzymatic activity increased five-fold in the liver of 3-month-old male CYB5R3-Tg mice fed SD. A-C, n=3 mice per group. Data are represented as the mean ± SEM. **p*< 0.05 compared to Wt animals. (E) Body weight and (F) average daily calorie intake of CYB5R3-Tg and Wt mice fed either SD or HFD for 17 weeks. (G) Daily calorie intake divided by body weight over the same period. (H) Glucose levels after a 20-h fasting period in HFD-fed mice. (I) Serum insulin levels in HFD-fed mice. (J) HOMA-IR index in HFD-fed mice, n=5-7 per group. (K) Spontaneous activity of mice in the metabolic chambers, n=4-6 per group. (L) Rotarod performance in HFD-fed mice. (M-O) Interscapular and whole body temperature in anesthetized 24-month old mice: (M) Fed mice; (N) mice fasted for 16 h; and (O) oral gavage of glucose (1.5 g.kg^-1^ body weight) after 6-h fasting, n=4 per group. Unless otherwise stated, data are shown for all the animals as assigned by the protocol design detailed in panel 2G. Data are represented as the means ± SEM. **p*< 0.05 compared to diet-paired Wt groups.

**Figure S3, Related to Figure 3. Ingenuity pathway analysis (IPA) of canonical pathways in response to CYB5R3 overexpression in the livers of SD-fed mice**

**Figure S4, Related to Figure 3. IPA’s canonical pathway within the category of “Xenobiotic P450 pathway” in CYB5R3-Tg mice fed SD**

**Figure S5, Related to Figure 4. Representative MRI images of livers from CYB5R3-Tg and Wt mice included in the DEN protocol**

Representative MRI images acquired with a MSME pulse sequence with fat suppression (echo time 12.5 ms). The left side of the mouse is shown on the right side of each image and the ventral side of the mouse is at the top, per radiological conventions. (Scale bar, 0.5 cm; n=4-10 per group). Segmentation of whole liver and liver tumors is indicated by light blue and dark blue color, respectively.

**Figure S6, Related to Figure 5. Liver lipidomic analysis**

Percent of the indicated fatty acid (FA) species in the livers of 5-month-old CYB5R3-Tg and Wt mice fed SD, n=4 per group. Data are represented as means ± SEM. **p*< 0.05 compared to Wt mice.

**SUPPLEMENTARY TABLES**

**Table S1, Related to Figure 2. List of major pathologies identified at necropsy**

| Organs | Pathology | Genotype | |  |
| --- | --- | --- | --- | --- |
|  |  | Wt | CYB5R3-Tg | |
| Heart | Enlarged | 6 (15.8%) | 6 (10.9%) | |
|  | Carcinoma | 0 (0%) | 0 (0%) | |
| Lungs | Enlarged | 3 (7.9%) | 3 (5.5%) | |
|  | Carcinoma | 2 (5.3%) | 3 (5.5%) | |
| Liver | Enlarged | 7 (18.4%) | 14 (25.5%) | |
|  | Carcinoma | 6 (15.8%) | 8 (14.5%) | |
| Spleen | Enlarged | 9 (23.7%) | 14 (25.5%) | |
|  | Carcinoma | 6 (15.8%) | 8 (14.6%) | |
| Kidneys | Enlarged | 7 (18.4%) | 6 (10.9%) | |
|  | Carcinoma | 2 (5.2%) | 0 (0%) | |
| Seminal Vesicles | Enlarged | 33 (86.8%) | 44 (80%) | |
|  | Carcinoma | 0 (0%) | 0 (0%) | |
| Bladder | Enlarged | 6 (15.8%) | 4 (7.3%) | |
|  | Carcinoma | 0 (0%) | 0 (0%) | |

n=38 for Wt and n=55 for CYB5R3-Tg mice, respectively. The percentage of mice per group is presented between brackets.

**Table S2, Related to Figure 3. List of top 25 up- and down-regulated GO terms in liver of CYB5R3-Tg vs. Wt mice fed SD**

| Gene Ontology Term | Z_Score |
| --- | --- |
| CYB5R3-Tg : WT |
| GO0004930 G PROTEIN COUPLED RECEPTOR ACTIVITY | 12.97 |
| GO0007186 G PROTEIN COUPLED RECEPTOR PROTEIN SIGNA | 12.56 |
| GO0007608 SENSORY PERCEPTION OF SMELL | 12.4 |
| GO0004984 OLFACTORY RECEPTOR ACTIVITY | 12.34 |
| GO0004871 SIGNAL TRANSDUCER ACTIVITY | 11.21 |
| GO0006694 STEROID BIOSYNTHETIC PROCESS | 9.79 |
| GO0004872 RECEPTOR ACTIVITY | 9.23 |
| GO0007165 SIGNAL TRANSDUCTION | 8.15 |
| GO0006695 CHOLESTEROL BIOSYNTHETIC PROCESS | 7.8 |
| GO0016021 INTEGRAL TO MEMBRANE | 7.8 |
| GO0016126 STEROL BIOSYNTHETIC PROCESS | 7.51 |
| GO0001584 RHODOPSIN LIKE RECEPTOR ACTIVITY | 6.44 |
| GO0005550 PHEROMONE BINDING | 6.19 |
| GO0042157 LIPOPROTEIN METABOLIC PROCESS | 6.02 |
| GO0016491 OXIDOREDUCTASE ACTIVITY | 5.9 |
| GO0008610 LIPID BIOSYNTHETIC PROCESS | 5.5 |
| GO0016020 MEMBRANE | 5.34 |
| GO0005840 RIBOSOME | 4.91 |
| GO0005743 MITOCHONDRIAL INNER MEMBRANE | 4.89 |
| GO0005843 CYTOSOLIC SMALL RIBOSOMAL SUBUNIT (SENSU | 4.84 |
| GO0003735 STRUCTURAL CONSTITUENT OF RIBOSOME | 4.68 |
| GO0016503 PHEROMONE RECEPTOR ACTIVITY | 4.41 |
| GO0004252 SERINE TYPE ENDOPEPTIDASE ACTIVITY | 4.4 |
| GO0019236 RESPONSE TO PHEROMONE | 4.32 |
| GO0005830 CYTOSOLIC RIBOSOME (SENSU EUKARYOTA) | 4.24 |
|  |  |
| GO0043433 NEGATIVE REGULATION OF TRANSCRIPTION FAC | -4.06 |
| GO0008219 CELL DEATH | -4.07 |
| GO0003677 DNA BINDING | -4.14 |
| GO0005524 ATP BINDING | -4.17 |
| GO0007264 SMALL GTPASE MEDIATED SIGNAL TRANSDUCTIO | -4.18 |
| GO0006898 RECEPTOR MEDIATED ENDOCYTOSIS | -4.23 |
| GO0015031 PROTEIN TRANSPORT | -4.24 |
| GO0006334 NUCLEOSOME ASSEMBLY | -4.39 |
| GO0042605 PEPTIDE ANTIGEN BINDING | -4.5 |
| GO0005575 CELLULAR COMPONENT | -4.59 |
| GO0007001 CHROMOSOME ORGANIZATION AND BIOGENESIS ( | -4.65 |
| GO0019886 ANTIGEN PROCESSING AND PRESENTATION OF E | -4.71 |
| GO0006350 TRANSCRIPTION | -4.99 |
| GO0006512 UBIQUITIN CYCLE | -5.01 |
| GO0007049 CELL CYCLE | -5.12 |
| GO0007242 INTRACELLULAR SIGNALING CASCADE | -5.23 |
| GO0003676 NUCLEIC ACID BINDING | -5.48 |
| GO0005764 LYSOSOME | -5.77 |
| GO0001558 REGULATION OF CELL GROWTH | -5.87 |
| GO0000166 NUCLEOTIDE BINDING | -6.18 |
| GO0000074 REGULATION OF PROGRESSION THROUGH CELL C | -6.4 |
| GO0005622 INTRACELLULAR | -6.58 |
| GO0005737 CYTOPLASM | -6.72 |
| GO0005634 NUCLEUS | -8.75 |
| GO0005515 PROTEIN BINDING | -9.08 |

All GOTerms were statistically significant with Z Score >1.5 in both directions, p < 0.05 and false discovery rate < 0.3 (see Supplemental Experimental Procedures for additional details).

**Table S3, Related to Figure 5. Ingredients of AIN-93G chow**

| Ingredient | g.kg^-1^ | Kcal.kg^-1^ |
| --- | --- | --- |
| Casein, High Nitrogen | 200 | 716 |
| L-cysteine | 3 | 12 |
| Sucrose | 100 | 400 |
| Cornstarch | 397.1 | 1429.5 |
| Dextrose | 132 | 501.6 |
| Soybean Oil | 70 | 630 |
| t-Butyl hydroquinone | 0.014 | 0 |
| Cellulose | 50 | 0 |
| Mineral Mix#210025 | 35 | 30.8 |
| Vitamin Mix#310025 | 10 | 38.7 |
| Choline Bitartrate | 2.5 | 0 |

AIN-93G purified rodent diet (DYET#180823, Revised). Ingredients for food composition are shown as g.kg^-1^ and kcal.kg^-1^.

**Table S4, Related to Figure 5. Lipid composition of soybean oil in AIN-93G chow.**

| Soybean Oil | Value (% of weight) |
| --- | --- |
| Cholesterol | Trace |
| Myristic (14:0) | Trace |
| Palmitic (16:0) | 10.2 |
| Palmitoleic (16:1) | Trace |
| Stearic (18:0) | 4.5 |
| Oleic (18:1) | 22.7 |
| Linoleic (18:2) | 54.8 |
| Linolenic (18:3) | 7.8 |

Percentage by weight and fatty acid profile are shown. Values shown are percentage by weight of soybean oil.

**Table S5, Related to Figures 2-5. Primer pair sequences used for quantitative RT-PCR analysis**

| **Gene** | **Forward primer sequence** | **Reverse primer sequence** |
| --- | --- | --- |
| **MsCYB5R3** | 5’–TTTGTGGACTTGGTGGTCAA–3’ | 5’–CGGAATTCAATGGTGTCTCC–3’ |
| **MsIL1β** | 5’–GCAACTGTTCCTGAACTCAACT–3’ | 5’–ATCTTTTGGGGTCCGTCAACT–3’ |
| **Msp21** | 5’–CACAGCTCAGTGGACTGGAA–3’ | 5’–ACCCTAGACCCACAATGCAG–3’ |
| **MsRELA** | 5’– GGAGGGATCTTCGGTAGTGG –3’ | 5’– CCCTGCGTTGGATTTCGTG –3’ |
| **MsGAPDH** | 5’–CACCAACTGCTTAGCCCC–3’ | 5’–TCTTCTGGGTGGCAGTGATG–3’ |
| **MsGADD45** | 5’–ggaccctgacaatgtgacct–3’ | 5’–Agcagaacgcctgaatcaac–3’ |
| **MsSREBP1c** | 5’–tggaggcagagagcagagat –3’ | 5’–tcacaggttccccatagaca–3’ |
| **MsACCα** | 5’–aacatccccacgctaaacag –3’ | 5’–agtcacagaagcagcccatt –3’ |
| **MsACCβ** | 5’–tttctgatgtgctggaatgg –3’ | 5’–gactgtgtgtgctcgtggtt –3’ |
| **MsGPAT** | 5’–actgggttgactgtgggttc –3’ | 5’–gcaggaatagcagcaccttc –3’ |
| **MsFASN** | 5’–gcataacggtctctggtggt –3’ | 5’–gcggtgtgaaaacgaacttt –3’ |
| **MscJUN** | 5’– agcggatcaaggcagagag–3’ | 5’–ttttgcgctttcaaggtttt –3’ |
| **MsRps18** | 5’–TGTGTTAGGGGACTGGTGGACA –3’ | 5’–CATCACCCACTTACCCCCAAAA –3’ |
| **MsCOX2** | 5’–ATAACCGAGTCGTTCTGCCAAT –3’ | 5’–TTTCAGAGCATTGGCCATAGAA –3’ |
| **DrCYB5R** | 5’– AGTCGACAAGGCAAACGAAG –3’ | 5’– GGCGATCATTTCCTCATTG –3’ |
| **Drrp49** | 5’– CGGATCGATATGCTAAGCTGT –3’ | 5’– CGACGCACTCTGTTGTCG –3’ |

Ms: Mouse; Dr: *Drosophila Melanogaster*.

**SUPPLEMENTARY MATERIALS and METHODS**

**Animal maintenance and metabolic interventions**.

Mice were housed four per cage in a room maintained at a constant temperature (20°-22 °C) on a light:dark 12:12-h schedule according to animal protocols and NIH guidelines. Mice were maintained on *ad libitum* SD *(AIN-93G*) or HFD (diet D12492, Research Diets). Ingredients and lipid composition of SD chow are included in Tables S3 and S4. Body weight and food intake were measured every two weeks for the HFD study or monthly for the longevity study. The longevity study on the CYB5R3-Tg group started with 69 CYB5R3-Tg male mice and 64 Wt control male mice on the C57BL/6J background. Survival curves were plotted using the Kaplan-Meier method, which includes all available animals at each time point. Statistical analyses were performed using SigmaStat 3.5 (Systat Software Inc., San Jose, CA). The criterion for euthanasia was based on an independent assessment by a veterinarian, according to AAALAC guidelines, and only cases where the condition of the animal was considered incompatible with continued survival are represented in the curves. A total of two animals were censored in the lifespan study (1 Wt and 1 CYB5R3-Tg). For metabolic experiments, male mice were placed on either a SD or HFD *ad libitum* at 18-23 weeks of age. Sample sizes were: CYB5R3-Tg SD n=8, Wt SD n=15, CYB5R3-Tg HFD n=8, Wt HFD n=16.

Treatment of mice with tetrahydroindenoindole (THII) for microarray analysis was carried out as followed: Six- to-twelve-week-old female C57BL/6J mice were purchased from Jackson Laboratory (Bar Harbor, ME). Animals were group-housed, maintained on a 12-h light/dark cycle, and had access to rodent chow and water *ad libitum*. Mice were matched by initial body weight, and assigned to groups. Mice were maintained *ad libitum* on a normal diet that was pelleted, semi-purified, and nutritionally complete (AIN-93M, Dyets, Bethlehem, PA). For 10 weeks, mice drank plain water or water containing 100 µM THII. The consumption of water remained constant at 4 ml per day, such that the dosage per kg of body weight was about 4.5 mg THII/day. At the completion of the study, animals were euthanized and tissues were collected and processed for microarray analysis (see section below). Sample size: n=4 mice per group.

Treatment of mice with THII for longevity study was carried out as followed: Male C57BL/6J mice were obtained from Jackson Laboratory and aged in our colony, group-housed in cages of four with free access to diet and tap water *ad libitum*. For the longevity study, diet started at 113 weeks of age after randomization into two groups. Mice were fed a standard AIN-93G diet (Dyets) alone (n=14 mice) or supplemented with THII (n=16 mice). THII was added at a dose of 35 mg drug per kg of chow, formulated to provide daily doses of 2.7 mg drug.kg^-1^ body weight for the reminder of their lives.

Fly stocks were maintained on standard cornmeal agar medium at 25±1 ºC, 60±5% humidity and a 12:12h light/dark cycle. Gal4 driver da-Gal4 (w1118; P{w[+mW.hs]=GAL4-da.G32},3) was obtained from the Bloomington Drosophila Stock Center (Bloomington, Indiana). Flies were cultured on a sugar and yeast based diet (SY diet), which contains 2.5% sugar, 2.5% yeast and 1.5% agar. Lifespan assay was performed as previously described . Briefly, adult flies of mixed sex were collected within 24 h after eclosion and mated for 24 h. Flies were then sorted by sex, and placed in SY diet vials with approximately 20 male or female flies in each vial. Subsequently flies were transferred to vials with fresh food once every 2–3 days. Lifespan was measured by recording the number of dead flies at the time of each transfer until all flies were dead. 120-220 flies in 6-11 vials were used in each lifespan assay, which was repeated at least three times.

To attain Cyb5R overexpression in flies by RU486 treatment, UAS-CYB5R flies were mated with da-GSG driver flies on the cornmeal food. Flies that emerged within 24 h were collected and allowed to mate for 24 h in bottles with SY diet. Males and females were sorted out, placed in vials and fed SY diet for another 24 h. Flies then were transferred to SY diets with or without 200 μM RU486 for assays. Control flies and pre-induction flies were all maintained on the SY diet without RU486. Flies for stage-specific CYB5R-overexpression were transferred to SY diets supplemented with 200 μM RU486 on day 1 (young/healthy group), day 22 (transitional group) and day 43 (senescence group), respectively. Flies that completed stage-specific CYB5R-overexpression were switched back to regular SY diet without RU486 at day 22 (young/healthy group) and day 43 (transitional group). All flies were transferred to fresh diet (with or without RU486) once every 2-3 days, and the number of dead flies was recorded. For the lifespan assay, each vial contained approximately 20 flies. Lifespan of 100-200 flies in 6-10 vials was measured for each experimental group, which was repeated at least twice.

In another set of experiments, CYB5R OE and control flies were transferred to 50 ml vials containing 1 ml of a solid medium composed of 1.3% low melting agarose, 1% sucrose and 12 μM tunicamycin (all from Sigma) for ER stress test. The number of dead flies was recorded every 12 h.

**Quantitative RT–PCR**.

For mouse studies, total RNA was extracted from frozen liver samples from 35-40-week-old male mice using the RNeasy kit (Qiagen). Complementary DNA was synthesized from total RNA with the High Capacity cDNA reverse transcription kit (Applied Biosystems, Foster City, CA). For fly studies, heads from 14-day-old flies were isolated and used for total RNA extraction. Total RNA was extracted using Trizol reagent (Invitrogen Inc., Carlsbad, CA). cDNA was synthesized from total RNA using Superscript® reverse transcriptase from Invitrogen (Carlsbad, CA). The RT-PCR was performed on individual cDNAs by using SYBR® Green PCR master mix in the StepOne Plus Real-time PCR system (Applied Biosystems, Carlsbad, CA). The primer sequences are presented in Table S5. The mRNA expression was calculated by the 2^−ΔΔCT^ method and normalized to the expression of GAPDH for mice and *rp49* for flies.

# Cytochrome B5 reductase activity.

# CYB5R3 enzymatic activity was determined in protein lysates in 60 mM phosphate buffer, pH 7.5, 0.2 mM NADH, and 0.2 mM potassium ferricyanide. The reduction of ferricyanide was monitored at 420 nm at 25 ºC for 5 min. *N*-Ethylmaleimide at a concentration of 5 mM was used as a inhibitory control for CYB5R3 activity. CYB5R3 activity was calculated assuming an extinction coefficient of 1.02 mM^–1^.cm^–1^ .

**Food intake measurement in flies.**

A modified capillary feeder method (CAFE) was used to measure food intake as previously described . Briefly, two 12-day-old flies were housed in a feeding chamber with a feeding capillary filled with SY diet without 1.5% agar. Additional chambers with capillaries filled with SY diet but without flies were set up to account for the evaporation of liquid food. Food intake was measured once every 24 hours for three constitutive days. Daily food intake was calculated by averaging food intake in three days.

**Rotarod**.

Mice were given a habituation trial on day 1 in which they were placed on the rotarod at a constant speed (4 rpm) and animals remained on the rotarod for at least 1 min. Results shown are the average of 3 trials per mouse, measuring time to fall from an accelerating rotarod (4–40 rpm over 5 min). The maximum trial length was 5 min and there was a 30-min rest period between each trial.

**Insulin determination.**

To quantify insulin level, whole blood was obtained after a 20-h fast and spun at 14,000 rpm for 5 min to pellet blood cells. Plasma was transferred to a fresh tube, placed on dry ice and stored at -80 ºC. Plasma insulin levels were measured by using ELISA kits (Crystal Chem Inc., Downers Grove, IL).

**Homeostasis Model Assessment of Insulin Resistance (HOMA-IR)**.

Insulin resistance was estimated using the HOMA2 Calculator software available from the Oxford Centre for Diabetes, Endocrinology and Metabolism Diabetes Trials Unit website as described .

**Diquat assay.**

Acute inflammation was induced in 18-25-week old mice by an intraperitoneal injection of diquat at a dose of 60 mg.kg^-1^ body weight (Chem Service, West Chester, PA). Diquat was dissolved in isotonic saline and sterilized prior to injection. Animal survival was constantly monitored after the injection except for a 6-h overnight interval. Survival curves were plotted using the Kaplan-Meier method, which includes all live animals at each time point. Surviving mice were euthanized 24 h after injection.

**Paraquat assay.**

Adult flies were cultured on SY diet for 14 days, then transferred into vials containing 20 mM paraquat in 5% sucrose solution on 22-mm Waterman 3M filter discs. The number of dead flies was recorded once every 12 h. Each vial had approximately 20 flies. Six vials of flies were set up as replicates for each gender and genotype. Survival curves were plotted using the Kaplan-Meier method, which includes all live animals at each time point.

# ATP determination.

# Livers from 35-40-week-old male mice were homogenized in ATP-releasing buffer (100 m**M** potassium phosphate buffer at pH 7.8, 1% Triton X-100, 2 m**m** EDTA, and 1 m**M** dithiothreitol) for the determination of ATP levels using a luciferin-based ATP determination kit (Invitrogen, La Jolla, CA).

# NAD^+^/NADH determination.

# Livers from 35-40-week-old male mice were homogenized, and NAD^+^ and NADH levels were determined using a colorimetric-based determination kit (cat # AB65348, Abcam, Cambridge, MA).

# Acetyl-CoA determination.

# Livers from 35-40-week-old male mice were homogenized, and acetyl-CoA levels were determined using a fluorescence-based determination kit (cat # MAC039, Sigma-Aldrich, St. Louis, MO).

# Thermoimaging.

# Thermal image analysis was conducted using the Thermogenic Imaging ImagIR system (Millennium Pharmaceuticals). Fed, fasted (16 h) or glucose-gavaged (1.5 g.kg^-1^ body weight after a 6-h fasting) 24-month old mice (n = 4 per group) were anesthetized with ketamine hydrochloride/xylazine hydrochloride solution (1 µl.g^-1^ of body weight) and animals were placed in the thermoimager at 34 ºC. Temperature measurement was conducted in the interscapular region and across the whole body for 30 min. Data presented represent the average temperature during the 30-min period.

**Magnetic resonance imaging.**

Immediately following sacrifice, each mouse was placed in a Bruker Biospec 7T/30cm MRI scanner (Bruker Biospin, Ettlingen, Germany) equipped with a 72-mm resonator coil (Bruker). The mouse was inserted in a prone, feet-first position in a home-built bed and cooled with a stream of 4 °C air supplied by a vortex tube (AirTX, Cincinnati, OH) to retard decomposition during scanning. To detect and quantify liver tumors, a T2-weighted spin echo Multi-slice Multi-Echo (MSME) pulse sequence was used to acquire 10 contiguous, 0.75 mm thick slices with an in-plane resolution of 430 µm × 273 µm (left-right × anterior-posterior). For each slice, four echoes were acquired with echo time TE = 12.5 ms, 25 ms, 37.5 ms and 50 ms, respectively. Other parameters included repetition time TR = 1.5 s, number of signal averages NA = 2, spectral bandwidth SW = 100 kHz and total scan time of 6 min 24 s. From two to five contiguous packages of 10 slices each were required to cover the entire cranial-caudal extent of the liver, depending on its size. These scans were performed both without and with fat presaturation, which consisted of a 2.6 ms, 90° Gaussian pulse followed by a 2 ms, 40 mT/m spoil gradient. Finally, all scans were repeated with acquisition using a fat-suppressed fast spin echo (RARE) pulse sequence with RARE factor = 4, effective echo time TEeff = 26.6 ms and total scan time 1 min 36 s. Images were exported to ImageJ 1.47 (NIH, Bethesda, MD) and each set of two to five slice packages covering the liver were concatenated to produce a single 4D (MSME) or 3D (RARE) data set. For each slice, liver tumors were identified by hyperintensity relative to liver parenchyma. Distinguishing tumors from normal liver and fat was also facilitated by comparison of the four images acquired with incremented T2 weighting as well as with and without fat suppression. Contours were then manually drawn around the periphery of the liver as well as each contiguous tumor mass. The total liver area and total tumor area were calculated by counting the pixels within these contours. This analysis was repeated for each liver-containing slice and then the number of pixels in each slice was separately added for liver or tumor and multiplied by the volume of one pixel (0.088 µl) to yield total volumes.

**Body composition.**

Measurements of lean tissue, fat and fluid mass in whole live mice were performed at 72-89 weeks of age. The assessment was performed by nuclear magnetic resonance (NMR) using the Minispec LF90 (Bruker Biospin, Billerica, MA).

**Southern blot**.

Southern blotting was performed by Taconic (Germantown, NY). A ~360-bp probe was used for hybridization after plasmid digestion with the restriction enzymes MluI and SnaBI to confirm the presence of the transgene. EcoRV was used to digest the mouse genomic DNA prior to hybridization.

**Metabolic clamps.**

Mouse metabolic rate was assessed by indirect calorimetry in open-circuit Oxymax chambers using the Comprehensive Lab Animal Monitoring System (CLAMS; Columbus Instruments, Columbus, OH). Mice were housed singly with *ad libitum* access to water and food, and maintained at 20-22 °C under a 12:12 h light-dark cycle (light period 0600-1800). All mice were acclimatized to monitoring cages for 3-6 h prior to recording. Constant airflow (0.6 L.min^-1^) was drawn through the chamber and monitored by a mass-sensitive flow meter. The concentrations of oxygen and carbon dioxide were monitored by sensors placed at the inlet and outlet of the sealed chamber to calculate oxygen consumption as the difference between oxygen concentration in air entering the chamber and oxygen leaving the chamber. The sensor was calibrated against a standard gas mix containing defined quantities of oxygen, carbon dioxide and nitrogen. Data from each chamber were recorded for 30 s at 30-min intervals for a total of 60 h. The second dark:light cycle is represented in the plots. Movement (both horizontal and vertical) was also monitored. The system has light beams 0.5 inches apart on the horizontal plane, providing a high-resolution grid covering the XY-planes and the software provides counts of beam breaks by the mouse during 30-s intervals.

**Western blot analysis**.

Unless otherwise stated, Western blot analyses were performed in liver lysates from 35-40-week-old male mice. Samples were lysed in radioimmunoprecipitation buffer supplemented with ethylenediaminetetraacetic acid and ethylene glycol tetraacetic acid (Boston BioProducts, Ashland, MA), and the Bradford assay method (Bio-Rad, Hercules, CA) was used to determine protein concentration. Proteins were separated by [sodium dodecyl sulfate](http://en.wikipedia.org/wiki/Sodium_dodecyl_sulfate) [polyacrylamide](http://en.wikipedia.org/wiki/Polyacrylamide) [gel electrophoresis](http://en.wikipedia.org/wiki/Gel_electrophoresis) under reducing conditions and then transferred to nitrocellulose membranes. Western blots were performed according to standard methods, which involved a blocking step in 5% bovine serum albumin and incubation with a primary antibody of interest, followed by incubation with a horseradish peroxidase-conjugated secondary antibody. The visualization of immunoreactive bands was performed using the ECL Plus Western blotting detection system (GE Healthcare, Piscataway, NJ). In this study, the primary antibodies were directed against HADHSC (Cat#:74650), PGC1α/β (Cat#:13067), and GAPDH (Cat#:32233) (Santa Cruz Biotechnology, Santa Cruz, CA); IDH2 (Cat#:12652), pNF-κβ (Cat#:3033), pSTAT3 (Cat#:9131) and SIRT3 (Cat#:5490S) (Cell Signaling Technology, Beverly, MA); ACC (Cat#:04-322) and p-ACC (Cat#:05-373) (Millipore, Bedford, MA); SDHA (Cat#:MS204), UQCRC2 (Cat#:MS304), and ATP5A (Cat#:MS507) (MitoSciences, Eugene, OR); β-actin (Cat#:AB6276), NDUFA9 (Cat#:AB14713), SOD2 (Cat#:AB13533), acetyl K68-SOD2 (Cat#:AB137037), VDAC1 (Cat#:AB15895), NDUFB8 (Cat#:AB110242) and ACAA2 (Cat#:AB140529) (ABcam, Cambridge, MA); NF-κB (Cat#:1546-1) (Epitomics, Burlingame, CA); CYB5R3 (Navarro et al., 1995) and STAT3 (Cat#:610190) (BD Transduction labs, Lexington, KY). Antibodies were generally used at a dilution recommended by the manufacturer. Blots were quantified with ImageJ software and the bands of interest were normalized to the corresponding lanes in gels stained with Ponceau S, as previously validated by our group . Membranes were reprobed for β-actin and/or GAPDH to confirm equal loading in the gels.

**Mitochondrial enzymatic activities.**

Mitochondrial activities were determined in approximately 50 µg of liver lysates from 35-40-week-old male mice according to published methods . Activities of NADH:coenzyme Q oxidoreductase (complex I), succinate dehydrogenase (complex II), decylubiquinone: cytochrome *c* oxidoreductase (complex III), NADH:cytochrome c oxidoreductase (complex I to III) and succinate:cytochrome *c* oxidoreductase (complex II to III) were determined by spectrophotometric methods and normalized to citrate synthase activity present in the lysates.

**Coenzyme Q_9_H_2_ and coenzyme Q_9_ levels and redox ratio.**

DEN (Sigma-Aldrich) was injected intraperitoneally in 5-month-old male mice (100 mg.kg^-1^ of body weight). 48 h later, animals were sacrificed and livers were immediately dissected and frozen in liquid nitrogen. Total coenzyme Q_9_ was extracted from the liver according to a previously published method . In brief, frozen livers were homogenized in 20 mM potassium phosphate, pH 7.5, supplemented with 0.5 mM 2-mercaptoethanol, in a 1:15 volume-to-weight ratio, by using a pellet pestle motor (Sigma-Aldrich). 330 µl of n-propanol were added to 100 µl of homogenized liver and each sample was vortexed. After a 3-min incubation at room temperature, samples were vortexed and then centrifuged at 2500 × g for 5 min. 100 µl of supernatant was immediately injected into a high-performance liquid chromatographic apparatus equipped with a Spherisorb C-18 column (Sigma-Aldrich) operating at a flow rate of 1 ml.min^-1^ and an Electrochemical ESA Coulochem III detector. Coenzyme Q_6_ was used as internal control.

**Mitochondrial Isolation.**

Immediately following sacrifice, whole livers of 20-month old mice were excised and homogenized in a Teflon homogenizer at 40 RPM. Fresh mitochondria were isolated using the Mitochondrial Isolation Kit for Tissue according to the manufacturer’s instructions (Abcam).

**Mitochondrial membrane potential and superoxide determination**.

Twenty nM of tetramethylrhodamine, methyl ester (TMRM) (Invitrogen, Carlsbad, CA) or 3 µM MitoSOX (Invitrogen) were added to 10 µg of freshly isolated liver mitochondria from 20-month old mice. Samples were incubated in Mito buffer (105 mM K-MES, 30 mM KCl, 10 mM KH_2_PO_4_, 5 mM MgCl_2_.6H_2_O, 5 mg/ml BSA, 1 mM EGTA, pH 7.4) for 15 min (TMRM) or 30 min (Mitosox) at 37 °C. Fluorescence was measured by a flow cytometer (C6 Flow Cytometer, Accuri, MI) counting 50,000 events.

**Mitochondrial efficiency.**

Oxygen consumption in liver mitochondria from 20-month-old mice was determined with an Oxygraph-2k according the manufacturer’s instructions (Oroboros, Innsbruck, Austria). Oxygen consumption driven by specific respiratory-chain complexes was determined under state III conditions in freshly isolated liver mitochondria. Equal amounts of mitochondrial samples were placed in Mito buffer containing 1 mM ADP. Substrate concentrations were as follows: 10 mM pyruvate, 2 mM malate and 10 mM succinate. Reactions were terminated by addition of 10 mM cytochrome *c* to assess mitochondrial outer membrane integrity.

**Serum markers and hormones**.

Serum metabolites were quantified in serum from 25-week-old male mice that were fasted overnight using a COBAS Integra 400 instrument according to the manufacturer’s instructions (Roche, Indianapolis, IN).

**Superoxide production.**

Superoxide generation promoted by NADH and succinate addition to liver protein lysates from 35-40-week-old male mice was determined according to previously published methods . Results were expressed in nmol.mg protein^-1^.min^-1^.

**Microarray analysis.**

Liver RNA from 35-40-week-old male mice was isolated using the RNeasy kit (Qiagen, Valencia, CA) and then hybridized to BD-202-0202 Illumina Bead chips. Raw data were subjected to Z-normalization, as described elsewhere . Principal component analysis, performed on the normalized Z-scores of all of the detectable probes in the samples, was performed by using DIANE 6.0 software (<http://www.grc.nia.nih.gov/branches/rrb/dna/diane_software.pdf>). For the calculation of pairwise distances between samples, each microarray was considered as a point in a high-dimensional space since we treated each probe as a variable. Significant genes were selected by the z-test < 0.05, false discovery rate < 0.30, as well as z-ratio > 1.5 in both directions and ANOVA *P* value < 0.05. For parametric analysis of gene set enrichment (PAGE), our expression data was tested using the PAGE method as previously described . Briefly, for each pathway under a given pairwise comparison, an aggregated Z score was computed as:

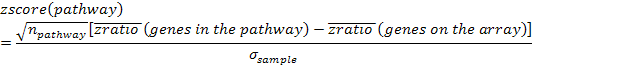

where
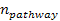
 is the number of genes in the specific pathway and
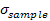
 is the standard deviation of z-ratio of the pairwise comparison in the current pathway. For each pathway, a *P* value was computed (JMP 6.0 software) from the total z-ratio in comparison with z-test. The false discovery rate (fdr) is also calculated based on the p-value for the whole pathway set as the multiple comparison correction. Ingenuity Pathways Analysis© was performed by using the tools supplied by Ingenuity Inc. (Ingenuity Systems; Redwood City, CA). All raw data are available in the Gene Expression Omnibus database (GSE50872). *n* = 4 per group; age = 35-40 weeks.

**Lipid peroxidation.**

Lipid peroxidation was determined in liver samples from 35-40 week-old male mice by quantifying the levels of 8-iso-prostaglandin F_2α_ using the 8-iso-PGF_2α_ELISA kit according to the manufacturer’s protocol (Enzo Life Sciences, Farmington, NY).

**Primary hepatocyte isolation.**

Primary hepatocytes were isolated from the livers of 4-month-old male mice (n=3 per group) as previously described . In brief, livers were perfused with liver perfusion medium (Invitrogen) using a peristaltic pump (Fisher Scientific). After exsanguination of the liver, perfusion was changed to Liver Digest Medium (Invitrogen) containing 50 μL of 150 mg.mL^-1^ collagenase I per 45 mL. Following centrifugation, cell pellets from three animals were pooled and were resuspended in DMEM supplemented with 10% FBS and antibiotics (penicillin/streptomycin) (Invitrogen) and seeded into 6-well plates precoated with collagen I. Cells that were attached overnight were used for various experiments.

**Palmitate oxidation.**

Palmitate oxidation was determined according to a previously published method with minor modifications . In brief, primary hepatocytes from CYB5R3-Tg and Wt mice were seeded in 6-well plates at a density of 1 × 10^6^ cells per well and incubated overnight. After a 30-min preincubation with 2 ml of palmitate oxidation assay buffer (POAB) (20 mM HEPES, pH 7.4, 114 mM NaCl, 4.7 mM KCl, 1.2 mM KH_2_PO_4_, 1.16 mM MgSO_4_, 2.5 mM CaCl_2_, and 1% fatty acid-free bovine serum albumin) supplemented with 3 mM glucose, cells were incubated with 30,000 cpm [1-^14^C] palmitate (*5* Ci/mol) (Perkin Elmer, Wellesley, MA), glucose, and L-carnitine at a final concentration of 3 or 20 mM and 0.8 mM, respectively. A paper filter with 150µl of 10N KOH was mounted in place in each well with 5/16-inch flexible anti-short bushing (cat # 292579; Home Depot, Apin Hill, MD) and plates were sealed using an optical adhesive film (Applied Biosystems, Rockville, MD). After a 2-h incubation at 37 °C, 100 μl of 7% perchloric acid (Sigma) was injected into each well and after an additional 2 h at 37 °C, the filters with captured ^14^CO_2_ were transferred into scintillation vials and the associated radioactivity was measured using liquid scintillation cocktail (Ecoscint, National Diagnostics, Inc., Charlotte, NC).

##### Glucose incorporation into lipids.

##### Primary hepatocytes were seeded in 6-well culture dishes at 1 x 10^6^ cells per well and were incubated overnight in DMEM supplemented with 10% FBS and antibiotics (penicillin/streptomycin). After a 60-min preincubation in POAB at 37 ºC, cells were treated with 30,000 cpm of [3-^3^H] glucose (0.5 Ci/mol, Perkin Elmer) and 30,000 cpm of [1-^14^C] palmitate (5 Ci/mol) (Perkin Elmer) and plates were sealed with an optical adhesive film. The [1-^14^C] palmitate was included as a tracer. The incubation was terminated 2 h later by adding 1 ml of methanol:PBS (2:3) to the cells. Cells were collected with gentle pipetting, centrifuged at 700 × **g**, and washed twice with PBS. Two hundred μl of 0.2 ***M*** NaCl was added to the cell pellet and the mixture was immediately frozen in liquid N_2_. The lipid and aqueous fractions were separated using a mixture composed of 750 μl of CHCl_3_:methanol (2:1) and 50 μl of 0.1***N*** KOH per sample. After vigorous vortexing, both phases were separated by centrifugation at 2000 × **g** for 20 min. The top aqueous layer was discarded, and the bottom lipid-soluble layer was washed with 200 μl of methanol:water:CHCl_3_ (48:47:3). Aliquots (200 μl) of the lipid-soluble phase were transferred into scintillation vials and radiolabeled lipids were quantified.

**Cholesterol determination.**

Cholesterol levels in liver tissue were determined using the Amplex Red Cholesterol Assay Kit according to the manufacturer’s instructions (Invitrogen).

**mtDNA to nDNA ratio analysis**.

Total DNA was extracted from the livers of 35-40-week old male mice with the DNeasy Blood and Tissue Kit (QIAGEN). mtDNA was amplified using primers specific for the mitochondrial cytochrome c oxidase subunit 2 (*Cox2*) gene and normalized to nuclear genomic DNA by amplification of the ribosomal protein s18 (*Rps18*) nuclear gene. Primers were designed using IDT software (IDT, Dallas, TX) and the primer sequences can be found in Table S5.

**SUPPLEMENTARY REFERENCES**
