# Supplementary figures and images for: Cytochrome b5 reductase and the control of lipid metabolism and healthspan
Source: NPJ Aging Mech Dis. 2016 May 12;2:16006–. doi: 10.1038/npjamd.2016.6 (PMC5515006; doi:10.1038/npjamd.2016.6)

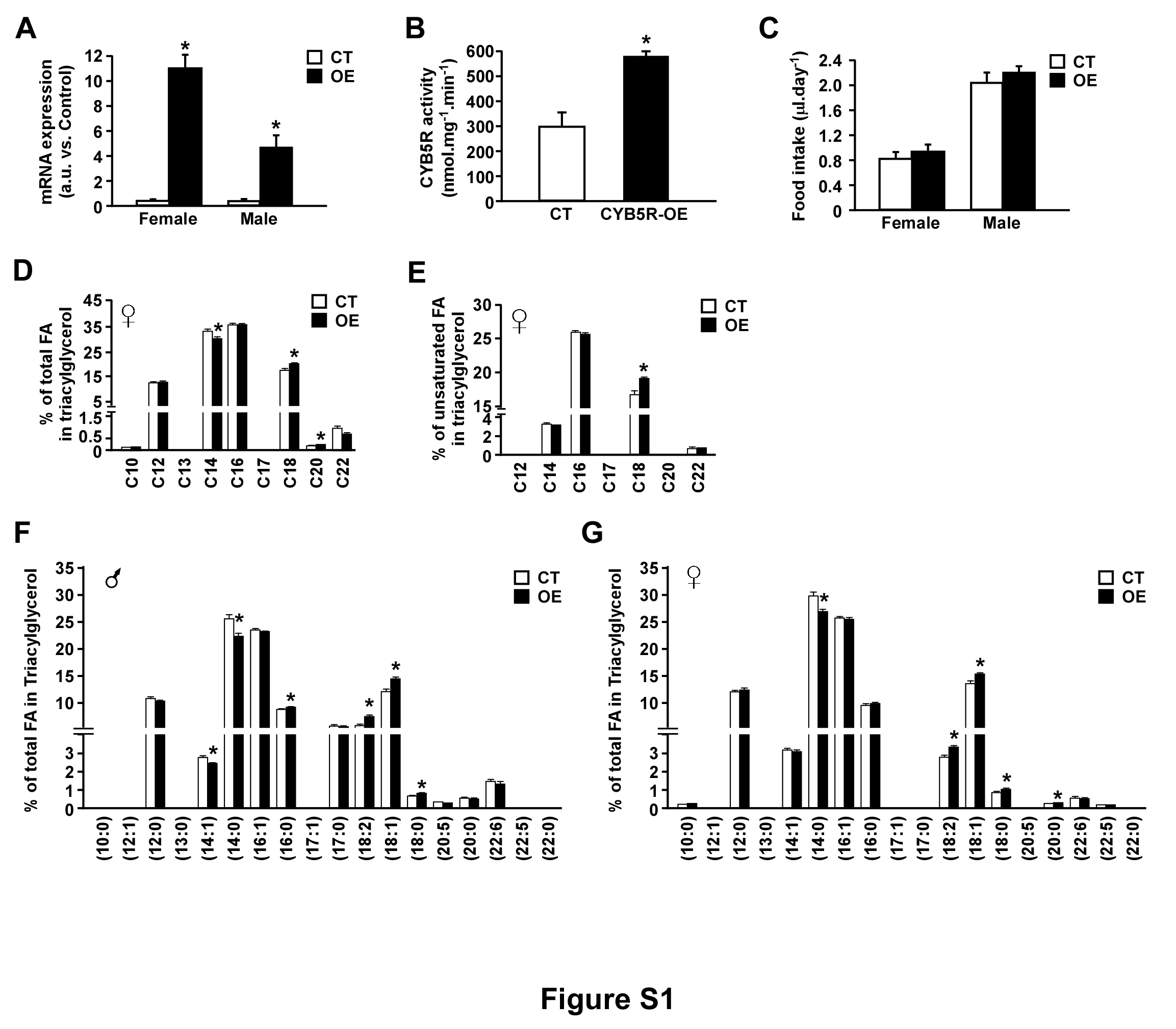

Supplement: Supplementary Figure S1 [file npjamd20166-s2.tiff]

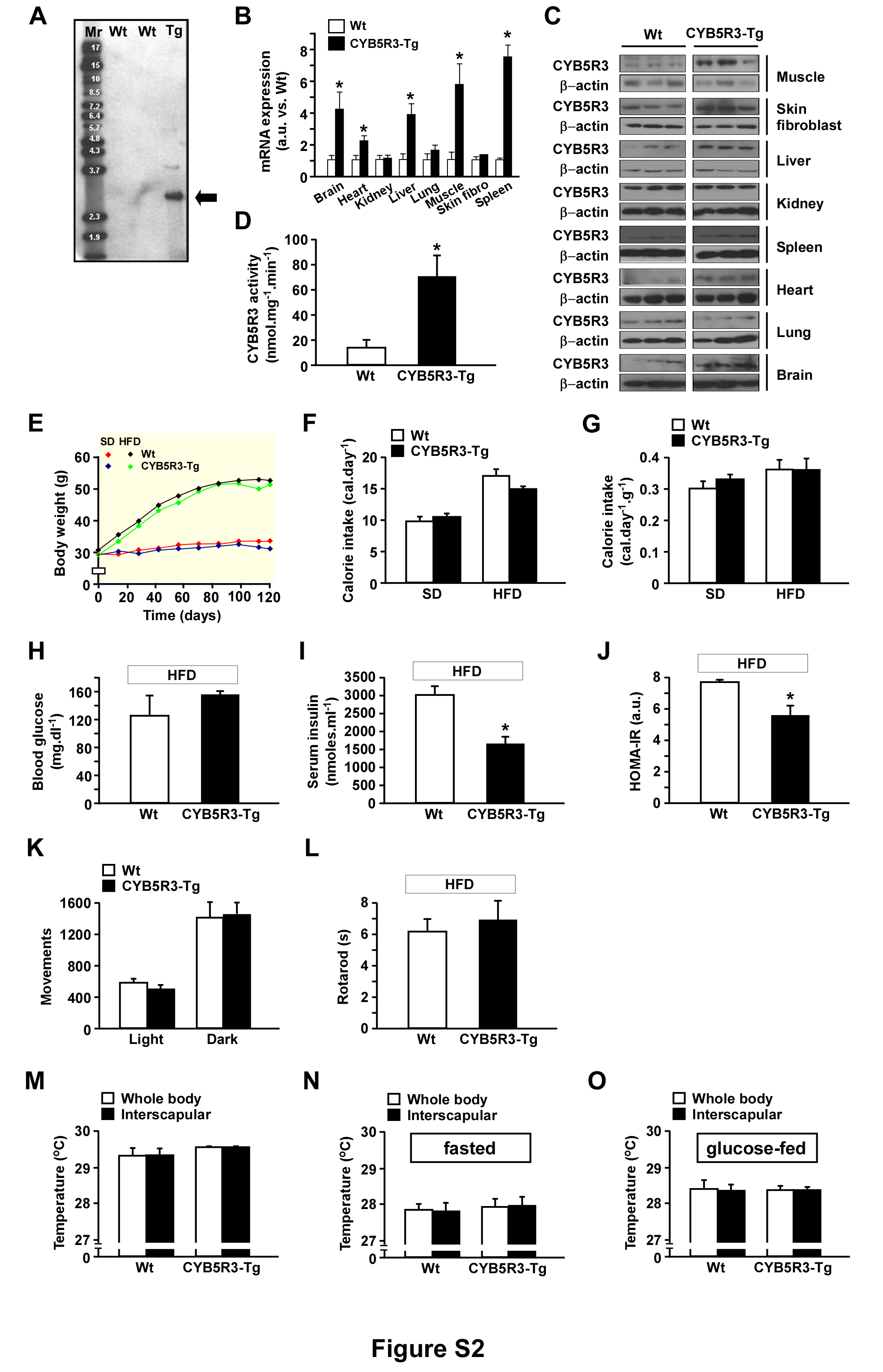

Supplement: Supplementary Figure S2 [file npjamd20166-s3.tiff]

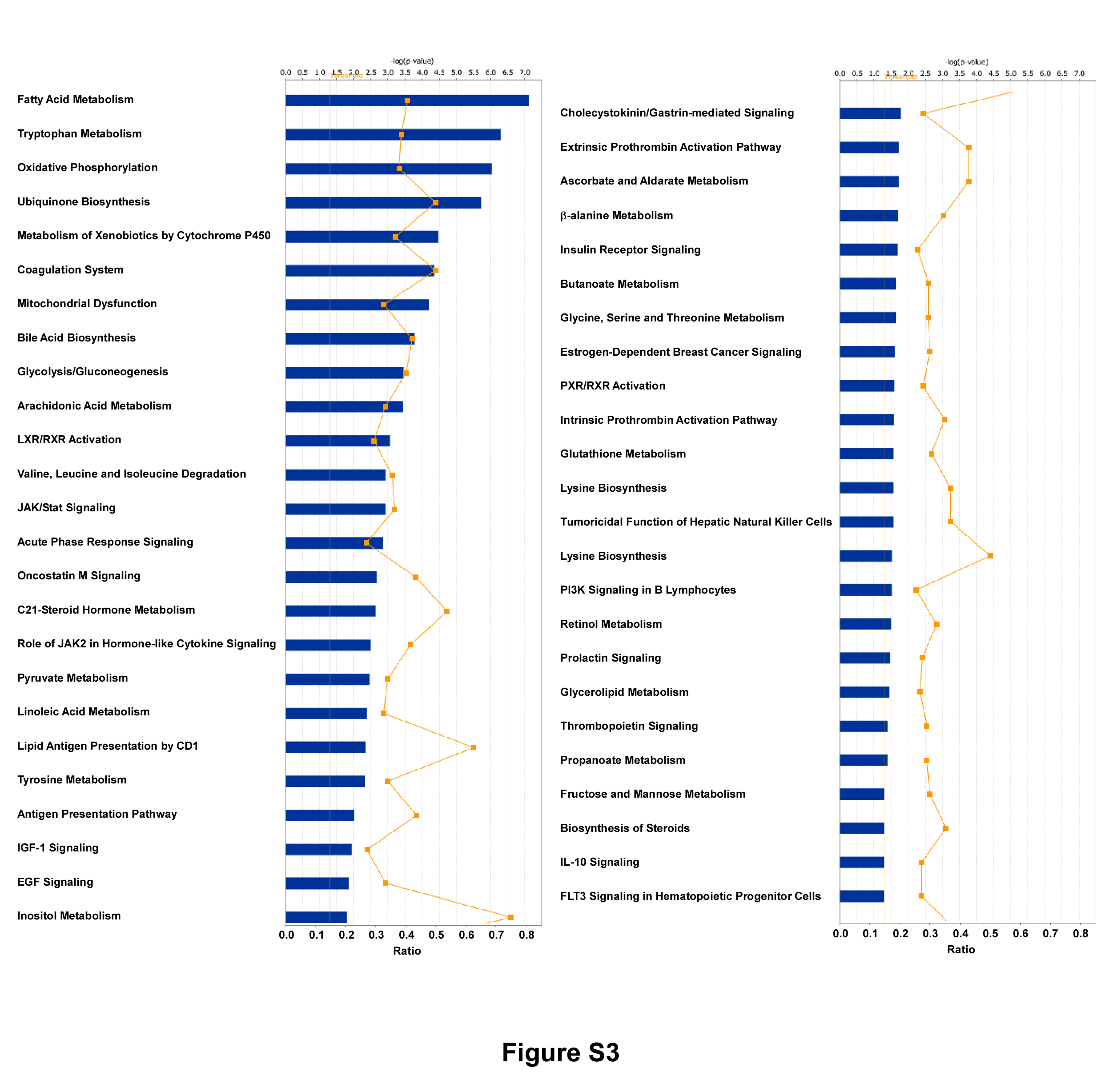

Supplement: Supplementary Figure S3 [file npjamd20166-s4.tiff]

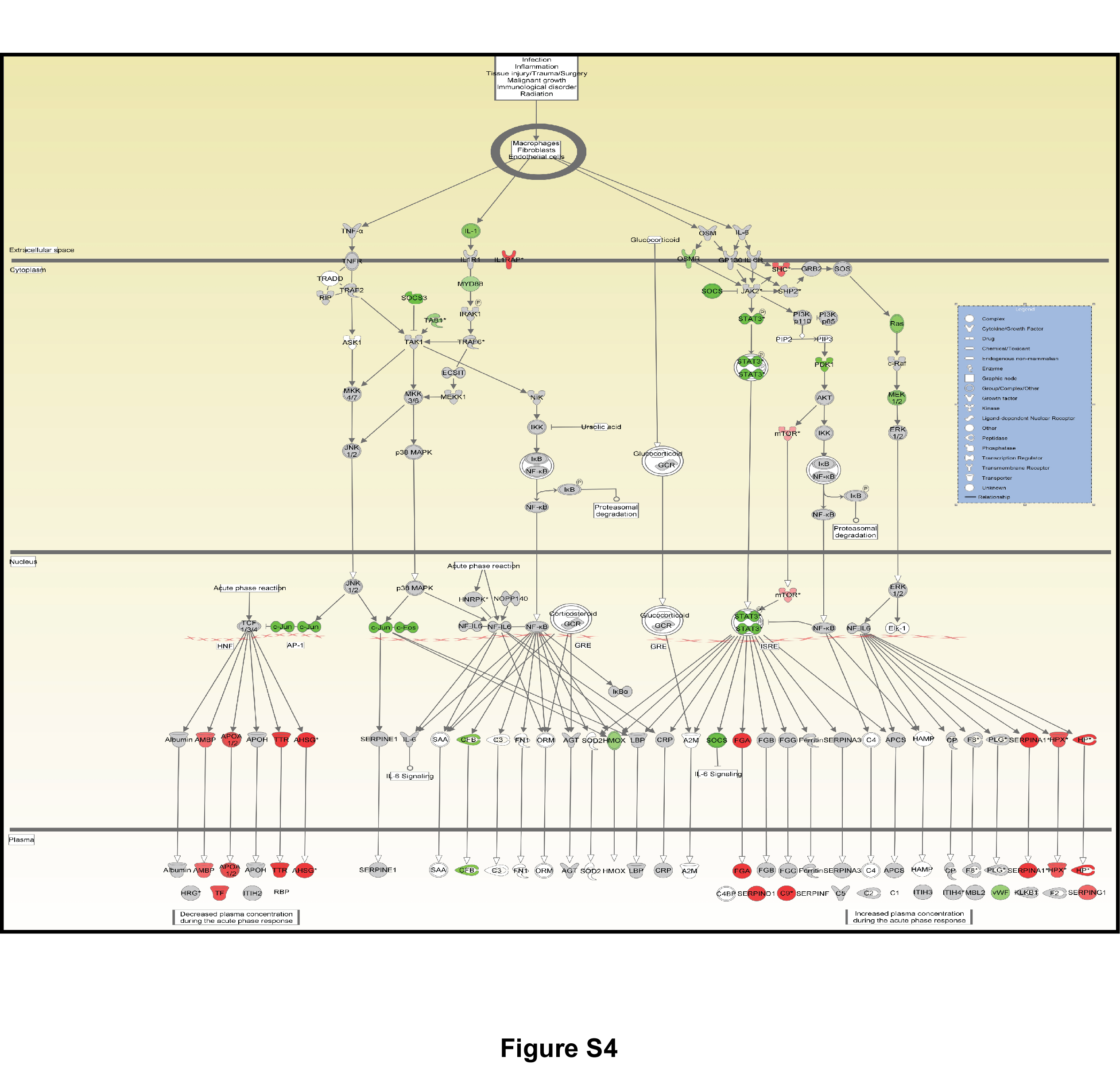

Supplement: Supplementary Figure S4 [file npjamd20166-s5.tiff]

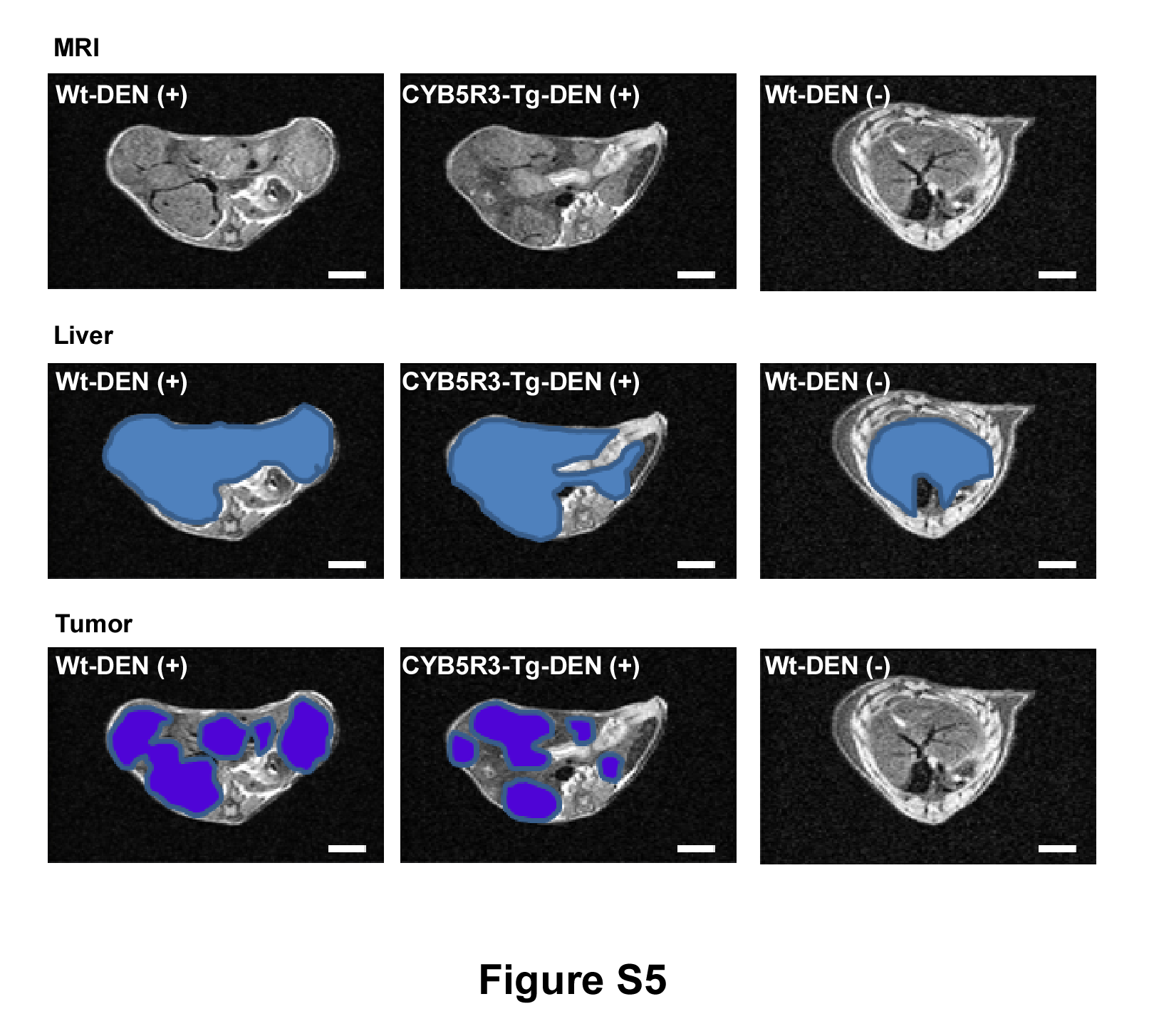

Supplement: Supplementary Figure S5 [file npjamd20166-s6.tiff]

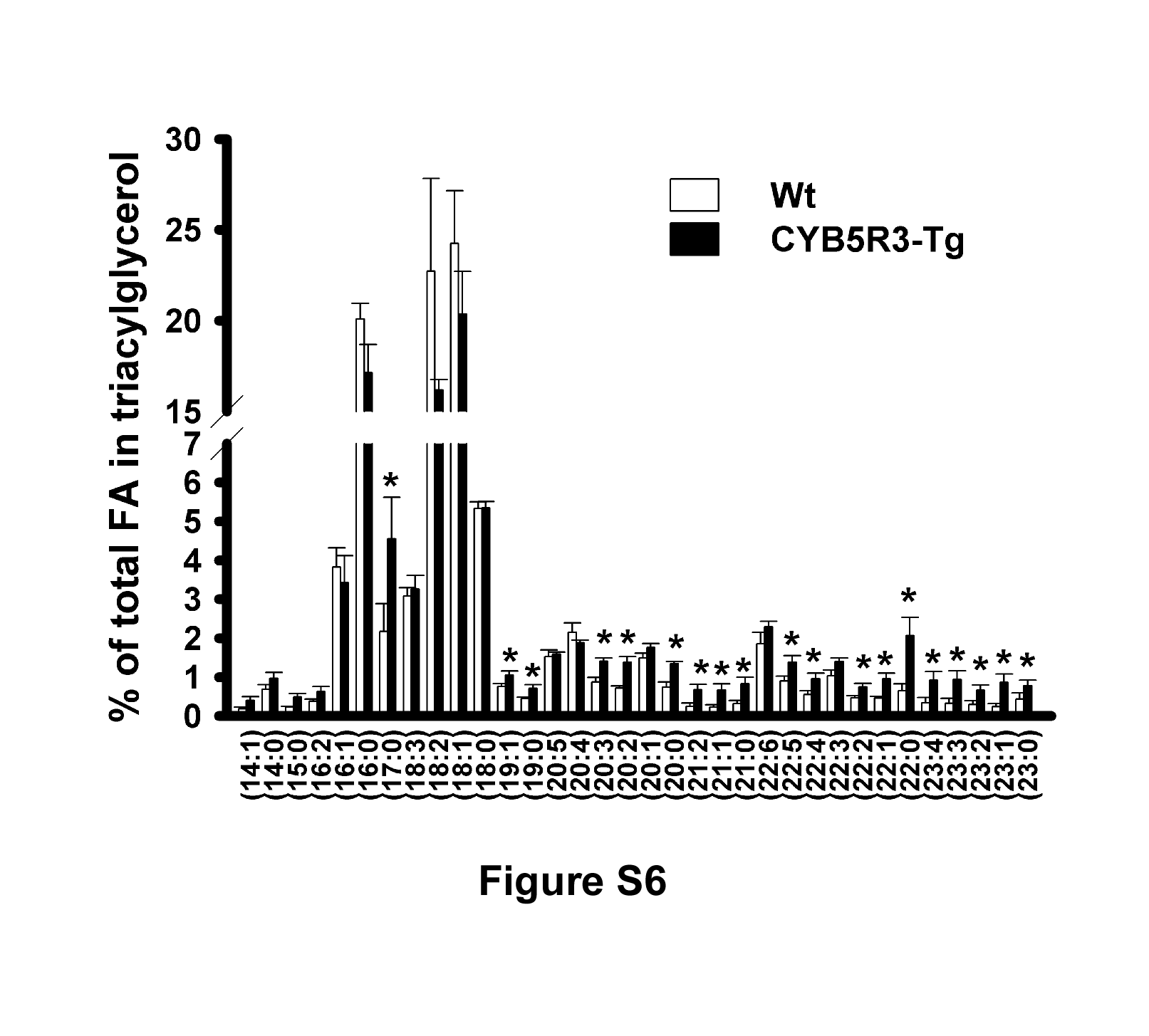

Supplement: Supplementary Figure S6 [file npjamd20166-s7.tiff]
